# Potentiating Cerebral Perfusion Normalizes Glymphatic Dynamics in Systemic Inflammation

**DOI:** 10.1002/advs.202503576

**Published:** 2025-10-05

**Authors:** Ruoyu Zhao, Bin Sun, Pengju Wei, Yingying Sun, Qianyan He, Kejia Zhang, Jun Lu, Shoujun Zhu, Yi Yang, Zhenni Guo

**Affiliations:** ^1^ Stroke Center Department of Neurology The First Hospital of Jilin University Chang Chun 130021 P. R. China; ^2^ Neuroscience Research Center Department of Neurology The First Hospital of Jilin University Changchun 130021 P. R. China; ^3^ Joint Laboratory of Opto‐Functional Theranostics in Medicine and Chemistry First Hospital of Jilin University Changchun 130021 P. R. China; ^4^ Institute of Biomedicine and Biotechnology Shenzhen Institutes of Advanced Technology Chinese Academy of Sciences Xueyuan Avenue 1068, Nanshan Shenzhen Guangdong 518055 P. R. China

**Keywords:** cerebral blood flow, glymphatic system, levosimendan, near‐infrared II fluorescence imaging, sepsis‐associated encephalopathy, systemic inflammation

## Abstract

The glymphatic system is essential for maintaining fluid homeostasis and clearing metabolic waste in the brain. Existing evidence suggests that this system is disrupted by systemic inflammation; however, the pathological characteristics and mechanisms underlying this disruption remain unclear. Near‐infrared II imaging reveals an excessive increase in perivascular cerebrospinal fluid (CSF) influx and a reduction in CSF efflux, accompanied by impaired glymphatic clearance within 72 h in mice intraperitoneally injected with lipopolysaccharide (LPS) at a dose of 5 mg kg^−1^, which does not significantly disrupt the blood‐brain barrier. An inverse relationship is observed between cerebral blood flow (CBF) and glymphatic influx trends following LPS challenge. Enhancement of CBF via levosimendan effectively ameliorates glymphatic flux and clearance, but these improvements are abolished by bilateral carotid artery stenosis surgery, indicating that cerebral hypoperfusion mediates LPS‐induced glymphatic dysfunction. Furthermore, levosimendan administration attenuates LPS‐induced neuroinflammation and neurological deficits. Mechanistically, CBF augmentation prevents the LPS‐induced perivascular Aquaporin‐4 (AQP4) depolarization, whereas the AQP4 inhibitor TGN‐020 blocks its beneficial effects on both amyloid‐β clearance and neuroinflammation suppression, confirming AQP4's pivotal role. Behaviorally, levosimendan ameliorates LPS‐induced neurological deficits. These findings establish cerebral hypoperfusion as a key mediator of systemic inflammation‐induced glymphatic dysfunction, revealing a promising therapeutic avenue for sepsis‐associated encephalopathy.

## Graphical abstract

LPS‐induced systemic inflammation increases glymphatic influx but delays cervical lymphatic drainage, accompanied by AQP4 depolarization and impaired glymphatic clearance. Enhancing cerebral blood flow via the inotropic agent levosimendan effectively restored AQP4 polarization, improving glymphatic flux and amyloid‐β clearance. Additionally, levosimendan broadly mitigates neuroinflammation and ameliorates LPS‐induced neurological dysfunction.

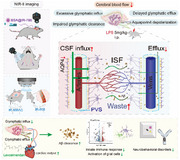

## 1 Introduction

Sepsis, a life‐threatening condition characterized by organ dysfunction, results from a dysregulated host response to infection.^[^ ^1^ ^]^ Sepsis can induce diffuse cerebral dysfunction, which ranges from mild delirium to coma and is collectively termed sepsis‐associated encephalopathy (SAE).^[^ ^2^ ^]^ SAE is highly prevalent, affecting ≈70% of patients with severe sepsis, and is associated with increased mortality and long‐term cognitive impairment.^[^ ^3^, ^4^ ^]^ Unlike encephalitis, which involves direct infection within the brain causing damage to the central nervous system (CNS), SAE is an indirect brain dysfunction triggered by infection in sepsis primarily via systemic inflammation.^[^ ^5^, ^6^ ^]^

The glymphatic system is an astrocytic endfeet‐covered perivascular network, which drives the exchange of cerebrospinal fluid (CSF) and interstitial fluid (ISF).^[^ ^7^, ^8^, ^9^ ^]^ Aquaporin‐4 (AQP4), the most abundant aquaporin in the brain, is highly polarized at astrocytic endfeet and serves as a key driver of the glymphatic system, mediating bidirectional CSF‐ISF exchange. Changes in AQP4 expression levels and its subcellular redistribution collectively represent key regulatory mechanisms governing its functional modulation.^[^ ^10^, ^11^, ^12^ ^]^ The glymphatic system mediates fluid transport through a highly compartmentalized yet interconnected anatomical pathway. In brief, CSF enters the brain parenchyma along the arteries, mixes with ISF, and exchanges solutes.^[^ ^13^ ^]^ This fluid mixture is then drained through the dural venous sinuses, meningeal lymphatic vessels, and cervical lymph nodes, ultimately entering the bloodstream via the subclavian vein.^[^ ^14^ ^]^ The process plays a critical role in clearing water‐soluble metabolic waste, facilitating immune surveillance, and maintaining fluid homeostasis.^[^ ^15^ ^]^

Defects in meningeal lymphatic vessels or ligation of the cervical lymphatic vessels result in the aggregation of pathological proteins, such as amyloid β (Aβ) plaques, tau tangles, and Lewy bodies, which are associated with neurodegeneration.^[^ ^16^, ^17^, ^18^ ^]^ In acute brain injuries such as traumatic brain injury, ischemic stroke, and hemorrhagic stroke, dysfunction of the glymphatic system exacerbates adverse outcomes.^[^ ^19^, ^20^, ^21^ ^]^ Acute systemic inflammation has been shown to disrupt glymphatic function;^[^ ^22^ ^]^ however, the temporal characteristics, underlying mechanisms of this disruption, and the relationship between glymphatic dysfunction and the progression of SAE remain unclear.

Under physiological conditions, the fluctuation of CSF flow is coupled with cerebral blood flow (CBF) rhythms and arterial pulsations, suggesting a regulatory role of CBF on the glymphatic system.^[^ ^23^, ^24^ ^]^ Diffuse cerebral ischemia is a significant event in the pathogenesis of sepsis.^[^ ^25^, ^26^, ^27^ ^]^ Therefore, we hypothesize that systemic inflammation‐induced hypoperfusion is an important cause of glymphatic dysfunction in sepsis. Levosimendan, a calcium‐sensitizing drug with positive inotropic and vasodilating properties, increases cardiac output without raising myocardial oxygen demand, and thus holds potential for improving glymphatic function by augmenting CBF.^[^ ^28^ ^]^ The loss of perivascular spaces (PVS) following animal death leads to the displacement of CSF tracers within these spaces, rendering traditional histological examination methods insufficient for accurately reflecting the function of the glymphatic system.^[^ ^29^ ^]^ To address this issue, we developed three Near‐infrared II (NIR‐II) fluorescence probes to mimic large and small molecular wastes in the brain for non‐invasive in vivo monitoring of CSF transport within the glymphatic system.^[^ ^30^, ^31^, ^32^ ^]^ In this study, we found that systemic inflammation induced by lipopolysaccharide (LPS) leads to dysfunction of the glymphatic system, characterized by excessive influx and impaired efflux. We further demonstrated that cerebral hypoperfusion is responsible for this dysfunction using levosimendan. The improvement of neuroinflammation by levosimendan highlights the therapeutic potential of targeting glymphatic function in SAE.

## 2 Results

### 2.1 LPS Bi‐phasically Alters Glymphatic Influx

**Figure 1:**
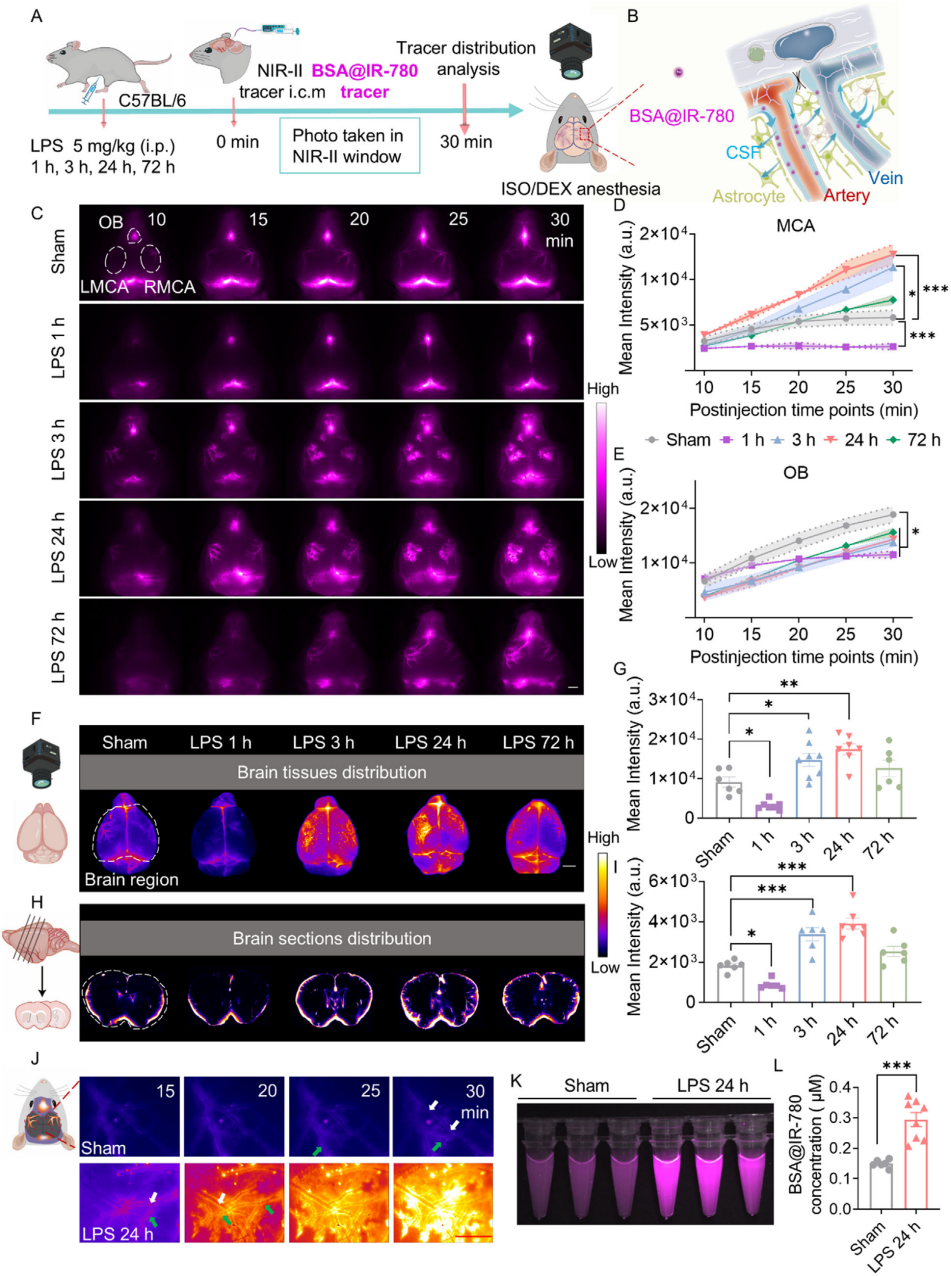
LPS alters glymphatic influx. A) NIR–II imaging of glymphatic influx following LPS injection under ISO/DEX anesthesia. B) BSA@‐IR780 mimics the perivascular CSF flow. (C) The representative in vivo NIR‐II images of CM‐ injection BSA@‐IR780 (CSF tracer) influx at 1, 3, 24, and 72 h after 5 mg kg^−1^ LPS i.p. injection, for 30 min, with a 5 min interval. The dotted white line delineates the area designated for quantification. White scale bar: 2 mm. *n* = 6–8/group. Mean pixel intensity in arbitrary units (A.U.) around MCA (D)/OB (E) for 30 min post‐CM injection. F,G) Representative images and quantitative analysis of BSA@IR‐780 distribution in the dorsal cerebrum of sham and LPS‐treated mice. Images were acquired at 1, 3, 24, and 72 h after LPS injection, 30 min post‐CM injection. The dotted white line delineates the area designated for quantification. White scale bar: 2 mm. *n* = 6–8/group. (H, I) Representative images and quantitative analysis of BSA@IR‐780 distribution in coronal brain sections. *n* = 6–7/group. J) Micro‐imaging of the perivascular CSF inflow in the NIR‐II window. Red scale bar: 1 mm. K) NIR‐II imaging of supernatants from brain homogenates collected 30 min post‐CM injection of BSA@IR‐780. *n* = 6–8/group. L) Quantitative analysis of BSA@IR‐780 concentration in the supernatants. Statistical analysis: D, E) Two‐way repeated‐measures ANOVA; (G, I) One‐way ANOVA; (L) Student's *t‐*test. Data are presented as means ± SEM. ^*^ *p* < 0.05, ^**^ *p* < 0.01 and ^***^ *p* < 0.001. White arrow: blood vessel. Green arrow: perivascular CSF.

We used NIR‐II imaging to noninvasively assess glymphatic system efficiency at different times after intraperitoneal LPS administration. We selected a biomimetic NIR fluorescent protein (FP) with a molecular weight of 66.9 kDa (BSA@IR‐780) to simulate CSF components. The BSA@IR‐780 FP was formed by inserting a Cl‐containing NIR dye into the protein pocket, followed by covalent binding between the C–Cl bond of the dye and the –SH group of the protein, resulting in greatly enhanced brightness and photostability.^[^ ^31^ ^]^ Glymphatic tracer inflow typically dominates within the initial 30 min, after which the signal begins to decrease, reflecting glymphatic efflux.^[^ ^33^, ^34^ ^]^ Specifically, we examined the CSF flow dynamics by slowly infusing 7 µL FP into the cisterna magna (CM) at 1 µL min^−1^, ensuring minimal perturbation of intracranial pressure.^[^ ^35^ ^]^ The distribution of the FP in the brain was monitored for 30 min after tracer injection under isoflurane (ISO)/dexmedetomidine (DEX) anesthesia, with a 5 min interval (**Figure** **1**A,B).

Wild‐type C57BL/6J mice were intraperitoneally injected with LPS to induce systemic inflammation. At 1 h post‐LPS (5.0 mg kg^−1^), the NIR‐II tracer intensity in the cortical middle cerebral artery (MCA) territory was significantly lower than that in the controls, indicating that glymphatic influx (CSF in the PVS around MCA cortical branches) was reduced (Figure 1C,D). However, the patterns of glymphatic influx were quickly reversed and maintained with elevations from 3 h after LPS injection. The NIR‐II images and the quantified fluorescence intensity showed that at 3 h and 24 h, the glymphatic influx activity progressively and significantly increased, and at 72 h, the glymphatic influx activity returned to the baseline level (Figure 1C,D). NIR‐II microscope provided clearly delineated vascular pathways and PVS contours, which could reflect more detailed elevated influx processes at 24 h following LPS administration (Figure 1J). The nasopharyngeal lymphatic plexus serves as the primary hub for CSF outflow to the cervical lymph nodes.^[^ ^16^ ^]^ NIR‐II imaging and quantitative analysis showed that olfactory fossa (OB) drainage was continuously reduced from 1 to 72 h after LPS injection (Figure 1C,E). Additionally, we found that a dose of 1.0 mg kg^−1^ LPS was sufficient to induce excessive glymphatic‐CSF influx in the mouse brain without significantly affecting OB drainage (Figure S1A–C, Supporting Information). To comprehensively investigate the damage of LPS to the glymphatic system, in the subsequent experiments, we induced systemic inflammation in mice using a dose of 5 mg/kg LPS. *Ex vivo* brain tissue harvested at 30 min post injection of BSA@IR‐780 validated our in vivo imaging findings, showing consistent NIR‐II tracer distribution in the dorsal cerebrum (Figure S2A,B, Supporting Information). The fluorescence intensity analysis of the NIR‐II tracer in both dorsal cerebrum and coronal sections aligned with the in vivo imaging results of glymphatic influx (Figure 1D,G,I). Notably, in coronal sections, we observed a distinct fluorescence gradient decreasing from the brain surface to deeper cortical layers, illustrating the CSF pathway from subarachnoid space along PVS into the brain parenchyma (Figure 1F–I; Figure S2C, Supporting Information). Furthermore, quantitative analysis of brain homogenates at 30 min post‐tracer injection revealed significantly elevated tracer levels in LPS‐treated mice, corroborating that LPS enhances glymphatic influx at 24 h post induction (Figure 1K,L).

The blood‐brain barrier (BBB) and the glymphatic system collectively regulate the transport of substances across the brain through complementary mechanisms.^[^ ^36^ ^]^ As reported, high‐dose LPS disrupts the integrity of the BBB.^[^ ^37^ ^]^ We assessed BBB integrity by conducting Evans Blue (EB) extravasation assay following 5 mg kg^−1^ LPS administration at 3, 24, and 72 h timepoints. Photothrombotic middle cerebral artery occlusion (pMCAO) model mice at 3 days post‐induction were utilized as positive controls. The results showed evident blue staining in pMCAO mouse brains, while no macroscopic blue coloration was observed in LPS‐treated brains (Figure S3A, Supporting Information). Quantitative analysis demonstrated significantly increased cerebral EB extravasation in pMCAO mice, whereas 5 mg kg^−1^ LPS maintained EB levels at baseline across the 72 h observation period (Figure S3B, Supporting Information). This finding was independently validated at 24 h by both 3‐kDa dextran permeability assays and endogenous IgG immunofluorescence (Figure S3C–F, Supporting Information), collectively suggesting this LPS dose is subthreshold for BBB disruption under our experimental conditions.

### 2.2 LPS Reduces Cervical Lymphatic Drainage

**Figure 2:**
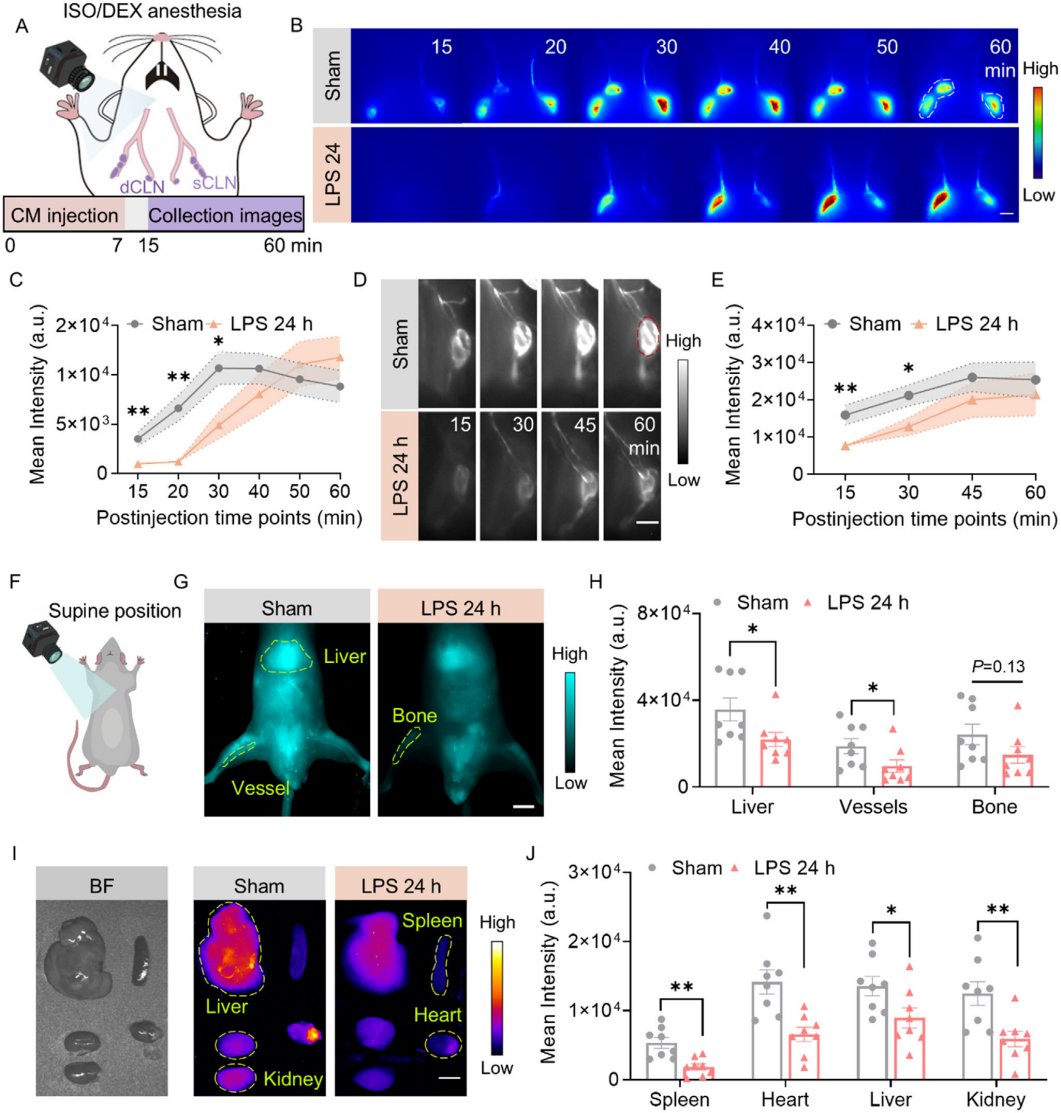
LPS reduces cervical lymphatic drainage. A) NIR‐II imaging of CSF drainage via cervical lymph nodes under ISO/DEX anesthesia. B) Representative images of in vivo sCLNs over 60 min for sham (gray) and LPS at 24 h (red). Time 0 marks the beginning of tracer injection, and imaging starts 15 min later. The dotted white line delineates the area designated for quantification (*n* = 6/group). White scale bar: 2 mm. C) Quantification of fluorescence intensity of sCLNs, analyzed by Student's *t‐*test. D) Representative in vivo NIR‐II images of dCLNs over 1 h with a 15 min interval (Sham, *n* = 6; LPS, *n* = 7). White scale bar: 1 mm. E) Quantitative analysis of fluorescence intensity of dCLNs, analyzed by Student's *t*‐test. F) Experimental outline for whole‐body imaging. G) Representative in vivo NIR‐II images of the whole body (ventral view) after monitoring the CLNs (*n* = 8/group). White scale bar: 1 cm. H) Quantitative analysis of fluorescence intensity of livers, vessels, and bones. Data were analyzed by Mann‐Whitney *U* test. I) Representative images of BSA@IR‐780 distribution in major excretory organs (spleen, heart, liver, kidney, *n* = 8/group). White scale bar: 5 mm. J) Quantitative analysis of fluorescence intensity of *ex vivo* organs. Data were analyzed using Student's *t*‐test. Data are presented as means±SEM. ^*^ *p* < 0.05 and ^**^ *p* < 0.01.

The CSF‐ISF mixture drains through meningeal lymphatics to the cervical lymph nodes and ultimately into the systemic drainage, a key pathway for clearance. Eventually, it integrates into the systemic circulation and is excreted by peripheral metabolic organs.^[^ ^38^ ^]^ The cervical lymphatic nodes, as the terminal accumulation points in CSF glymphatic drainage, are the classic sites for observing the efflux process of the glymphatic system.^[^ ^19^, ^39^ ^]^ As we observed, the decrease in CSF tracer fluorescence intensity in the olfactory bulb territory suggested an abnormality in the glymphatic efflux process caused by LPS (Figure 1C,E). We next investigated the cervical lymphatic system drainage of CSF to assess the alterations in the glymphatic efflux process induced by LPS. Specifically, we assessed the BSA@IR‐780 drainage in superficial cervical lymph nodes (sCLN) and deep cervical lymph nodes (dCLN) using NIR‐II imaging under ISO/DEX anesthesia (**Figure** **2**B,D). The fluorescence signal of the sCLNs was significantly lower in the LPS group than in the sham group within 30 min after CM injection. Compared to the sham control, LPS delayed drainage into the sCLNs, followed by a gradual increase and peak in BSA@IR‐780 coverage of the sCLNs in the LPS group, whereas the sham group showed a slow increase, peak, and decline. This change in the sham group was likely due to the BSA@IR‐780 in the sCLNs gradually entering the bloodstream (Figure 2B,C). The fluorescence signal of the sCLNs was higher in the LPS group than in the sham group 30 min after injection initiation. These results suggested that LPS delayed and slowed the drainage of the CSF tracer to the sCLNs and reduced its return to the bloodstream, resulting in excess accumulation of the BSA@IR‐780 tracer in the sCLNs.

Next, we created an imaging window to visualize the process of BSA@IR‐780 drainage in the dCLNs (Figure 2D). The results also showed a reduction in cervical lymph node drainage of BSA@IR‐780 within 30 min after CM injection (Figure 2E). The prolonged blood circulation and liver and bone accumulation properties of BSA@IR‐780 enabled visual assessment of glymphatic efflux outcomes following LPS injection through whole‐body imaging. After CLN imaging, we imaged the body‐wide distribution of CM‐injected BSA@IR‐780 (Figure 2G) and observed that LPS injection reduced tracer accumulation in the liver, bones, and vessels (Figure 2G,H). This reduction was further confirmed by peripheral organ imaging collected at 1 h after the CM injection (Figure 2I,J). Collectively, these findings demonstrated that LPS reduced glymphatic efflux.

### 2.3 LPS Suppresses Glymphatic Clearance

**Figure 3:**
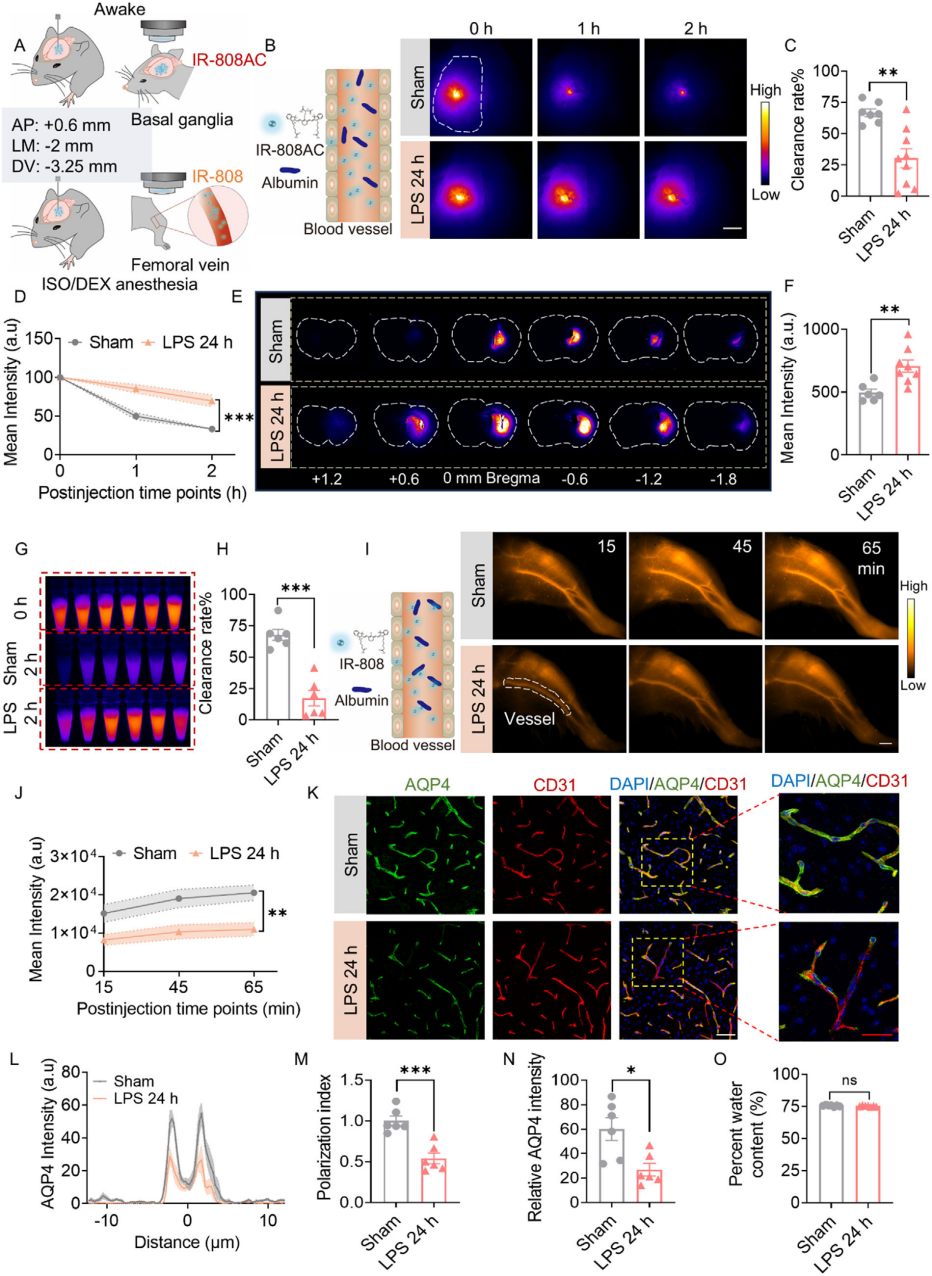
LPS reduces glymphatic clearance and perivascular AOP4 expression. A) Experimental flowchart of brain parenchymal waste clearance: IR‐808AC in conscious state; IR‐808 under ISO/DEX anesthesia. B) Left panel: IR‐808AC (NIR‐II cyanine dye) fails to bind with endogenous albumin. Right panel: Representative in vivo NIR‐II images of IR‐808AC fluorescence in brain at 0, 1, and 2 h after IR‐808AC infusion in the striatum, at 24 h post‐LPS injection. The dotted white circles delineate the area designated for quantification. *n* = 6–9/group. White scale bar: 2 mm. C) Analysis of clearance rate between sham and LPS groups. D) Quantification of fluorescence intensity within 2 h after IR‐808AC injection. E) Representative coronal sections (AP from +1.2 to −1.8 mm) depict the residual fluorescence of the NIR‐II tracer IR‐808AC. *n* = 6–9/group. F) Quantitative analysis of brain section fluorescence intensity. G) Brain tissues harvested at 0 h and 2 h post‐intrastriatal injection of IR‐808AC were homogenized and imaged under the NIR‐II window. *n* = 6/group. H) Quantification of clearance rate of IR‐808AC. I) IR‐808: A NIR‐II cyanine dye that binds with endogenous albumin. Representative IR‐808 images of the femoral vein within 65 min after striatal infusion. White scale bar: 2 mm. *n* = 6/group. J) Quantitative analysis of vessel fluorescence intensity. K) Representative AQP4 immunostaining images of the cortex at 24 h after LPS injection (green, AQP4; red, CD31; scale bars: 50 µm (white), 40 µm (red)). L) Cortical perivascular AQP4 intensity. M) Quantification of AQP4 polarization index. N) Quantitative analysis of AQP4 expression in the cortex, *n* = 6/group. O) Quantitative analysis of brain edema. Statistical analysis: D,J) Two‐way repeated‐measures ANOVA; C,F,H,M,N,O) Student's *t‐*test. Data are presented as means ± SEM.^*^ *p* < 0.05, ^**^ *p* < 0.01 and ^***^ *p* < 0.001.

The glymphatic system facilitates the CSF‐ISF exchange and ultimately transports neurotoxic substances and metabolites out of the brain. We next investigated glymphatic clearance at 24 h after LPS injection by injecting the NIR‐II tracer IR‐808AC into the striatum and assessing glymphatic clearance efficacy during wakefulness through in vivo imaging and brain sections (**Figure** **3**A–F). lR‐808AC, a NIR‐II albumin‐escaping probe, does not bind to proteins and is rapidly cleared with minimal residual fluorescence.^[^ ^30^ ^]^ These features enable the rapid clearance of lR‐808AC through glymphatic and cervical lymphatic transport within 2 h period. Figure 3B shows the representative in vivo images taken through the intact skull. The bright fluorescent signal in LPS‐treated mice indicated a marked reduction in glymphatic clearance, with the clearance rate reduced by ≈30% within 2 h (Figure 3B–D). The substantial accumulation of the NIR‐II tracer in the brain sections further verified that LPS reduced the glymphatic process of CSF‐ISF clearance (Figure 3E,F). In addition, we quantitatively determined the clearance rate of IR‐808AC by performing fluorometric analysis on brain homogenates. Brain tissues were collected immediately after probe injection completion (0 h) and 2 h post‐injection (Figure 3G). The clearance rate was calculated as [(Mean fluorescence intensity at 0 h ‐ Fluorescence intensity at 2 h) / Mean fluorescence intensity at 0 h] × 100%. Consistent with in vivo imaging results, LPS administration significantly impaired the probe clearance from the brain. At the 2 h time point, the LPS group exhibited only 17.39% ± 6.21% clearance compared to 67.87% ± 4.39% in controls (Figure 3G,H).

The albumin‐binding NIR‐II tracer IR‐808 exhibits prolonged persistence in systemic circulation, thus enabling the monitoring of its extent of efflux from the CNS by measuring its concentration in peripheral blood.^[^ ^32^ ^]^ IR‐808 was injected into the brain parenchyma, and fluorescence intensity in the femoral vein was examined under ISO/DEX anesthesia, which directly reflects the glymphatic clearance efficacy of IR‐808 from the brain (Figure 3A,I). Continuous imaging of the femoral vessels revealed a steady increase in the IR‐808 signal in the sham control group, whereas LPS significantly reduced the IR‐808 mean intensity at the corresponding time points. After 65 min, the concentration of IR‐808 in the LPS group was ≈47% lower than that in the sham group (Figure 3I,J). The above results provided further evidence for impaired glymphatic clearance caused by LPS. The PVS, formed by the outer wall of cerebral blood vessels and astrocytic end‐feet, constitutes the structural basis of the glymphatic system.^[^ ^40^ ^]^ The astrocytic end‐feet highly express the water channel protein AQP4, which provides the driving force for CSF‐ISF exchange.^[^ ^41^ ^]^ Our immunofluorescence staining results showed that the LPS decreased polarization AQP4 around the blood vessels (Figure 3K–M), Immunofluorescence intensity quantification and western blot analysis further confirmed that LPS‐induced perivascular AQP4 alterations were associated with the down‐regulated expression of AQP4 (Figure 3N; Figure S4A,B Supporting Information). Such LPS‐induced depolarization of perivascular AQP4 may underlie the observed glymphatic dysfunction.

AQP4 mediates cytotoxic edema in the early phase of acute brain injury and subsequently mitigates vasogenic edema in later stages, playing a pivotal role in edema regulation.^[^ ^7^ ^]^ Therefore, we employed the wet–dry weight method to measure brain water content and investigate whether LPS‐induced AQP4 depolarization correlates with cerebral edema formation. The results showed that LPS did not induce significant brain edema, indicating that LPS‐induced AQP4 depolarization did not lead to substantial changes in brain water content (Figure 3O). Furthermore, immunofluorescence staining of meningeal lymphatic vessels (LYVE‐1^+^) revealed that LPS treatment significantly increased their area, suggesting possible meningeal lymphatic hyperplasia (Figure S4C,D, Supporting Information).

### 2.4 Levosimendan Improves Cerebral Perfusion and Suppresses LPS‐Induced Excessive Glymphatic Influx

**Figure 4:**
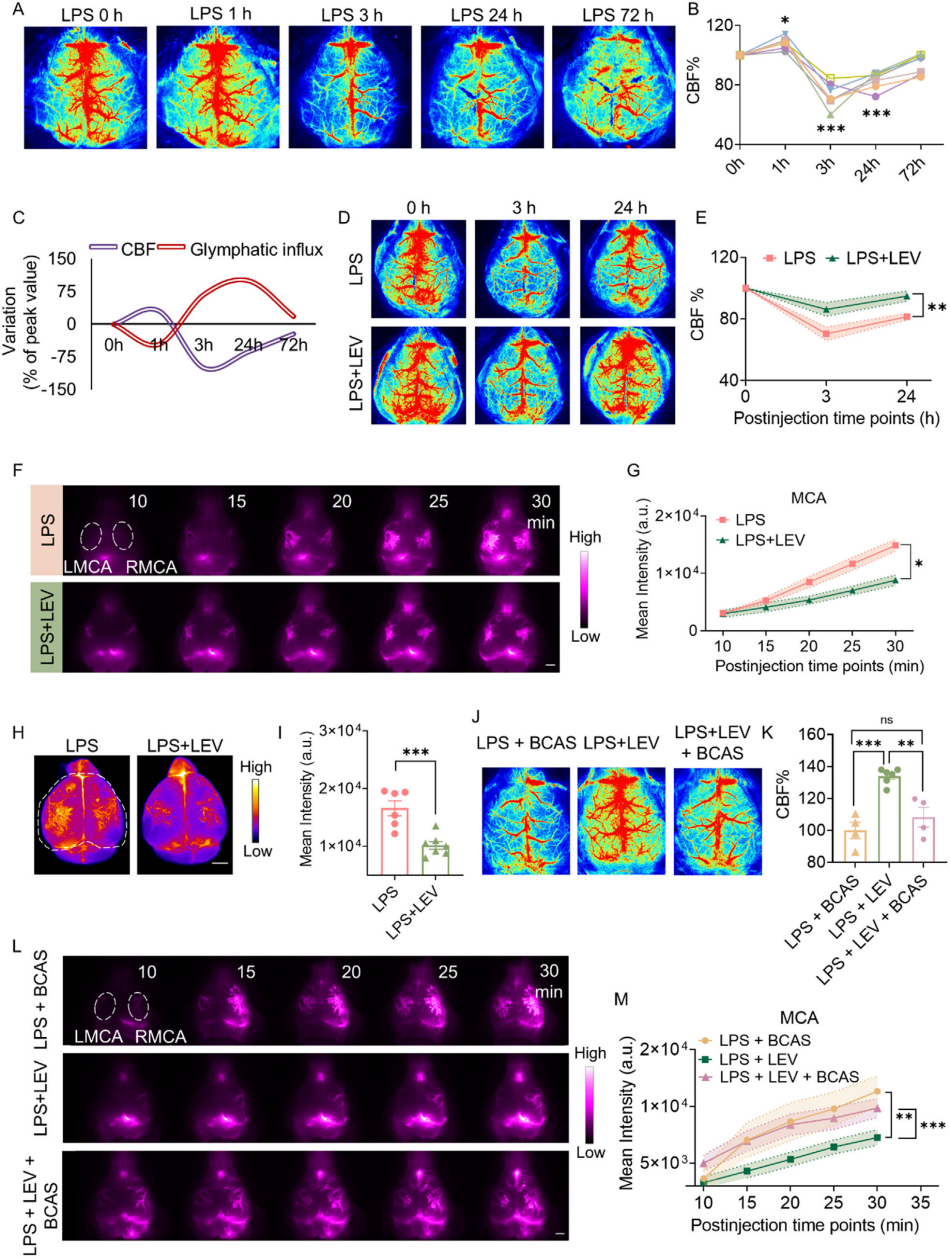
Levosimendan enhances CBF and reduces excessive glymphatic influx induced by LPS. A) Representative images of CBF by laser speckle in LPS treatment at 0, 1, 3, 24, and 72 h mice, where time 0 marks the point before LPS injection. *n* = 7. B) % quantification of CBF at 0–72 h, where each line represents an individual mouse (one‐way ANOVA). C) Temporal contrast of CBF versus glymphatic influx: smooth curves were generated by plotting amplitude‐normalized means (% of peak value). The mean intensity in the MCA territory is shown in Figure 1B. D) Representative images of laser speckle flowmetry post‐LPS injection. *n* = 6/group. E) Quantitative analysis of CBF. F) Representative in vivo images of BSA@IR‐780 influx (*n* = 7/group). White scale bar: 2 mm. G) Analysis of fluorescence intensity around the MCA. H) Representative images of BSA@IR‐780 distribution on the dorsal brain surface (*n* = 7), 30 min post‐CM injection. White scale bar: 2 mm. I) Quantitative analysis of fluorescence intensity over the dorsal brain. Representative images J) and quantitative CBF analysis K) 24 h after LEV treatment and subsequent BCAS. n = 4–6/group. L) Representative in vivo images of BSA@IR‐780 influx 24 h after LEV treatment and subsequent BCAS. (*n* = 5–6/group). White scale bar: 2 mm. M) Analysis of fluorescence intensity around the MCA. Statistical analysis: E,G,M) Two‐way repeated‐measures ANOVA; I) Student's *t‐*test; K) One‐way ANOVA. Data are presented as means ± SEM.^*^ *p *< 0.05, ^**^ *p *< 0.01 and ^***^ *p* < 0.001. Levosimendan (LEV).

Given that the glymphatic system is a perivascular network that facilitates CSF flow through tissues via the para‐arterial space and clears waste into the perivenous space, CBF responses to LPS may reversely regulate glymphatic influx. Laser speckle flowgraphy was employed to measure CBF. Comparative analyses between intact‐skull and skull‐removed conditions revealed highly congruent vascular networks in the dorsal cortical territory, validating the reliability of transcranial CBF measurement using laser speckle flowgraphy (Figure S5A,B, Supporting Information). Our results showed that the CBF increased slightly by ≈10% at 1 h post‐LPS, followed by a sharp decrease from 3 to 24 h post‐LPS, and then gradually recovered at 72 h after LPS (**Figure** **4**A,B). Amplitude‐normalized temporal contrast curves revealed an inverse dynamic relationship between CBF and glymphatic influx: an initial increase in CBF at 1 h post‐LPS corresponded with a decrease in perivascular CSF distribution, whereas a subsequent marked decline in CBF between 3 and 24 h was associated with the excessive glymphatic influx. Both parameters gradually returned to baseline levels by day 3 post‐LPS injection (Figure 4C).

Given the negative correlation between CBF and glymphatic influx, we hypothesized that cerebral hypoperfusion induces glymphatic dysfunction in systemic inflammation. To verify this hypothesis, we treated LPS‐injected mice with levosimendan. Levosimendan is a clinically approved inotropic agent that has the potential to increase CBF through enhancing cardiac output.^[^ ^42^, ^43^ ^]^ Our data showed that levosimendan normalized the LPS‐induced reduction in CBF from 3 to 24 h post‐LPS (Figure 4D,E). The NIR‐II images and quantified fluorescence intensity showed that at 24 h, levosimendan significantly suppressed the excessive glymphatic influx activity induced by LPS (Figure 4F–G). The racer distribution on the dorsal cerebral surface further confirmed the positive role of levosimendan in normalizing the LPS‐induced glymphatic influx (Figure 4H,I). Furthermore, the ameliorative effect of levosimendan on glymphatic influx was abolished when the increase in CBF was occluded by bilateral carotid artery stenosis (BCAS) induced by microcoils (Figure 4J–M). To strengthen evidence for the CBF‐glymphatic coupling, we employed digoxin as an additional perfusion enhancer to treat mice 0.15 mg kg^−1^ intraperitoneally at 1 h post‐LPS injection. Results demonstrated that digoxin simultaneously augmented CBF and reduced glymphatic influx (Figure S6A–D, Supporting Information). Moreover, linear regression analysis revealed a robust negative correlation between CBF and glymphatic influx (intensity of CSF tracer BSA@IR‐780 in the MCA territory at 30 min post CM‐injection (Figure S6E, Supporting Information).

Cerebral arterial pulsation is recognized as the principal driving force of perivascular CSF‐ISF exchange.^[^ ^29^, ^44^ ^]^ Our two‐photon in vivo imaging of the cerebral cortex revealed that LPS suppressed arterial pulsation, while levosimendan treatment attenuated the inhibitory effect of LPS on arterial pulsation (Figure S7A–E, Supporting Information). BCAS‐induced reduction in CBF, blocked the ameliorative effect of levosimendan on pulsation (Figure S7F–I, Supporting Information). We also evaluated the effects of levosimendan on other physiological parameters within 24 h post‐LPS injection, including cardiac function, peripheral blood pressure, and respiratory. Its administration mitigated the suppression of LPS on left ventricular ejection fraction (LVEF), fractional shortening (FS), heart rate, and mean arterial pressure (MAP), and alleviated LPS‐induced shortness of breath as well (Figure S8A–I, Supporting Information).

### 2.5 Levosimendan Antagonizes LPS‐Induced Reduction in Glymphatic Clearance and Cervical Lymph Drainage

**Figure 5:**
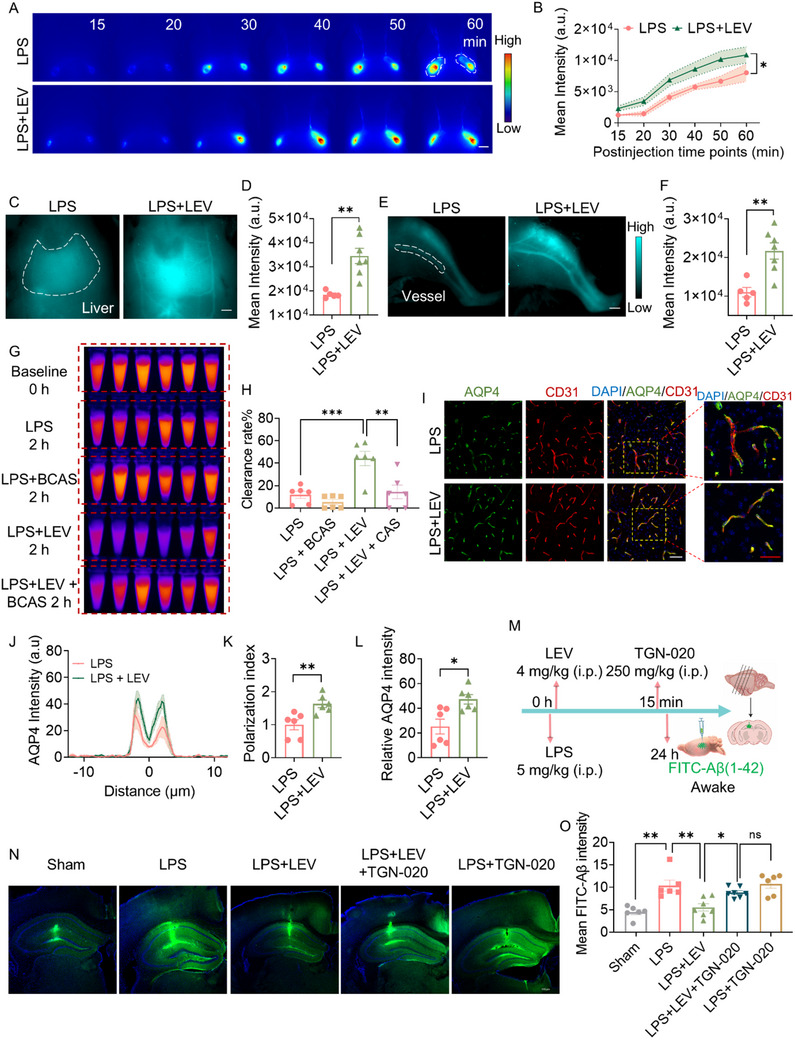
Levosimendan reverses LPS‐induced reduction in cervical lymph node drainage and glymphatic clearance. A) Representative images of BSA@IR‐780 distribution on sCLNs within 1 h post‐CM injection in the LPS (*n* = 5) and LPS + LEV (*n* = 7) treatment groups. White scale bar: 2 mm. B) Quantitative analysis of fluorescence intensity in sCLNs. Representative NIR‐II images of liver C) and vessels E) after monitoring sCLNs in the LPS and LPS + LEV treatment groups. *n* = 5–7/group. White scale bar: 2 mm C,E). Quantification of fluorescence intensity in liver D) and vessels F). G) Brain tissues harvested at 0 and 2 h post‐intrastriatal injection of IR‐808AC were homogenized and imaged under the NIR‐II window. *n* = 6/group. H) Quantification of clearance rate of IR‐808AC. I) Representative AQP4 immunostaining images of the cortex at 24 h after LPS injection (blue, DAPI; green, AQP4; red, CD31; white scale bar: 50 µm; red scale bar: 40 µm) J) Perivascular AQP4 intensity. K) AQP4 polarization index. L) Quantitative analysis of AQP4 expression in the cortex. *n* = 6/group. M) Experimental chart of FITC‐Aβ (1‐42) clearance in the hippocampus. Mice were allowed to recover from isoflurane anesthesia following intracerebral injection. N) Representative images of FITC‐Aβ (1‐42) distribution in the hippocampus were acquired 1 h post‐intrahippocampal injection (blue, DAPI; green, Beta‐Amyloid; scale bar: 500 µm). O) Quantitative analysis of fluorescence intensity of FITC‐labeled Aβ in brain. *n* = 6–7/group. Statistical analysis: B) Two‐way repeated‐measures ANOVA; D,F,K,L) Student's *t‐*test; H) One‐way ANOVA; (N) Kruskal‐Wallis test. Data are presented as means ± SEM. ^*^ *p* < 0.05, ^**^ *p* < 0.01 and ^***^ *p* < 0.001, as indicated.

Given the observed coupling between CBF and glymphatic influx, we then investigated whether enhancing CBF also could ameliorate LPS‐induced reduction in glymphatic clearance and CSF outflow via the cervical lymphatic system. The glymphatic efflux was assessed at 24 h after levosimendan administration. The sCLNs imaging showed that levosimendan increased cervical lymphatic drainage (**Figure** **5**A–B). Subsequently, we imaged the distribution of CM‐injected BSA@IR‐780 in the liver and femoral vessels and observed that levosimendan enhanced tracer accumulation in these areas (Figure 5C–F). The clearance assay of intraparenchymally injected IR‐808AC showed that levosimendan significantly enhanced its clearance from the awake brain at 24 after LPS induction, and this effect was blocked by BCAS, indicating that levosimendan enhances glymphatic clearance by augmenting CBF (Figure 5G,H).

Immunofluorescence 24 h after levosimendan administration showed a significant increase in the polarized perivascular distribution of AQP4 in the cortex compared to the LPS group (Figure 5I–L). Both fluorescence intensity measurements and Western blot analysis demonstrated that levosimendan treatment significantly promoted AQP4 expression (Figure 5J; Figure S9A,B, Supporting Information). The exchange of CSF‐ISF in the glymphatic system is critically dependent on AQP4 expressed on perivascular astrocytic end‐feet, which has been consistently demonstrated in multiple AQP4 knockout mouse lines.^[^ ^9^ ^]^ These data suggest an important role of perivascular AQP4 in levosimendan's improvement on LPS‐induced glymphatic dysfunction.

FITC‐labeled Aβ (1‐42) was injected into the hippocampus at 24 h after LPS induction and subsequently examining the fluorescence intensity of FITC in the awake brain at 1 h post‐intrahippocampal injection. Our findings indicate that levosimendan significantly attenuated LPS‐induced Aβ retention in the hippocampal territory (Figure 5N,O). TGN‐020, the most widely used astrocytic AQP4 inhibitor, was initially identified through combined molecular docking studies and Xenopus laevis oocyte swelling assays.^[^ ^45^, ^46^, ^47^ ^]^ Its inhibitory effects remain controversial due to potential artifacts in the oocyte swelling assay system and failure to replicate water transport inhibition in cellular experiments.^[^ ^48^, ^49^ ^]^ However, in vivo evidence still supports its function as an AQP4 inhibitor, albeit potentially through indirect mechanisms.^[^ ^50^, ^51^ ^]^ Out of caution, we first evaluated the effects of TGN‐020 on glymphatic function in naive mouse models. Our results demonstrate that intraperitoneal administration of TGN‐020 (250 mg kg^−1^) 15 min before BSA@IR‐780 CM injection effectively suppressed glymphatic influx and cervical lymphatic drainage, and decreased peripheral accumulation of BSA@IR‐780 in the liver and blood vessels, confirming its inhibitory effect on AQP4‐dependent glymphatic function (Figure S10A–H, Supporting Information).^[^ ^9^, ^46^, ^52^ ^]^ Similarly, TGN‐020 abolished levosimendan‐induced enhancement of FITC‐labeled Aβ clearance in the hippocampus, demonstrating that AQP4 repolarization is essential for levosimendan‐mediated improvement of glymphatic function (Figure 5N,O). Unlike its effects under physiological conditions, TGN‐020 failed to further reduce either perivascular influx or drainage of BSA@IR‐780 in LPS‐treated mice at 24 h post‐induction (Figure S10A–H, Supporting Information). This observation is consistent with the absence of significant differences in FITC‐labeled Aβ (1‐42) clearance efficiency among the three experimental groups: LPS, LPS+TGN‐020, and LPS+LEV+TGN‐020, suggesting that LPS‐induced AQP4 suppression may have precluded any additional inhibitory effect of TGN‐020 (Figure 5N,O).

Furthermore, levosimendan had no effect on the LYVE‐1 immunofluorescence area but showed a trend toward ameliorating the LPS‐impaired meningeal lymphatic OVA‐647 drainage at 30 min post‐CM injection of the tracer under ISO/DEX anesthesia (Figure S11A,B, Supporting Information). That indicates that levosimendan's rescue effect operates independently of structural remodeling in this downstream drainage pathway. Collectively, these data reveal that CBF enhancement rescues LPS‐induced glymphatic dysfunction through perivascular AQP4 repolarization.

### 2.6 Levosimendan Hampered LPS‐Induced Neuroinflammation

**Figure 6:**
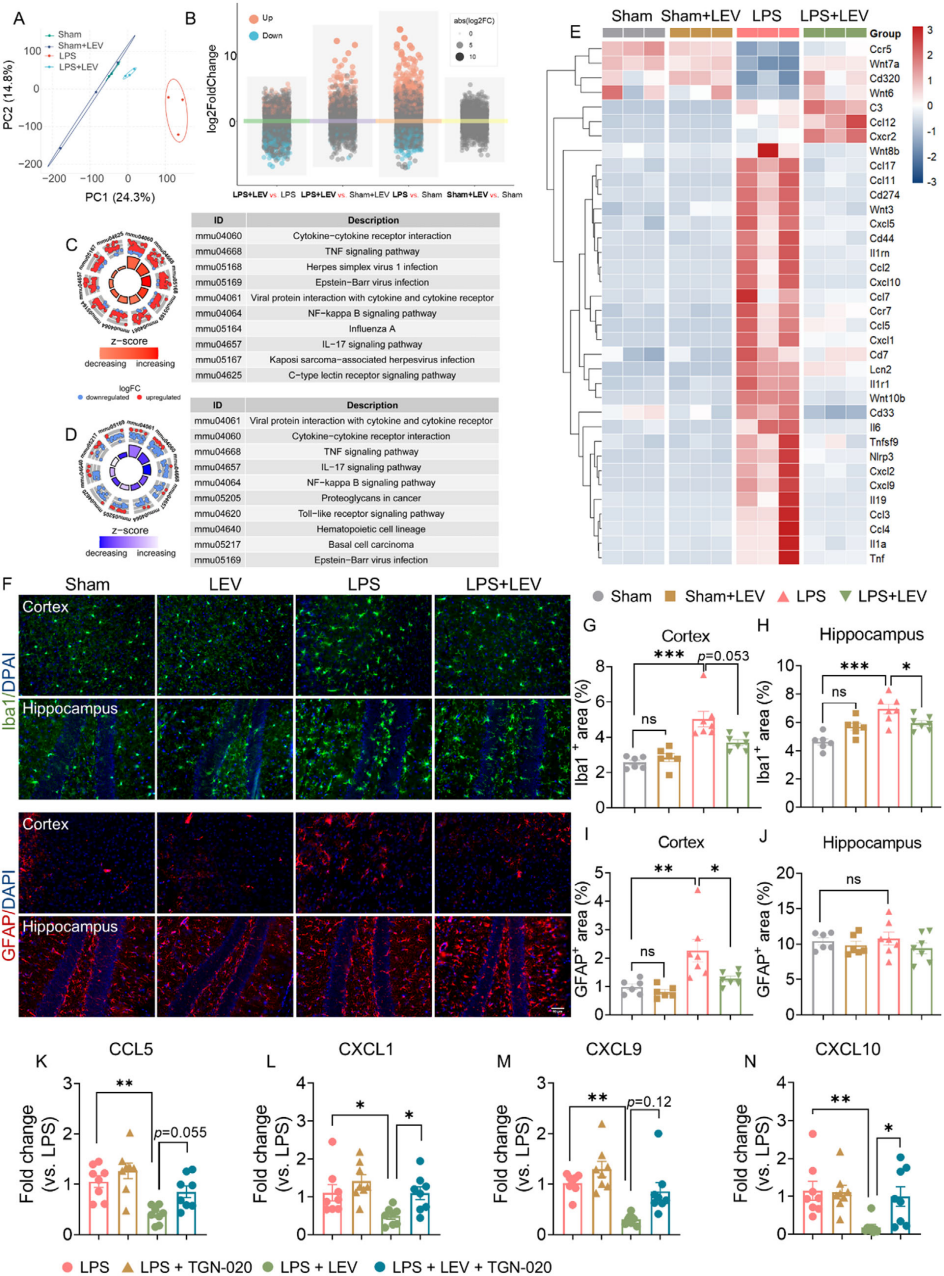
Levosimendan ameliorates neuroinflammation 24 h after LPS. A) PCA plot. *n* = 3 biological replicates/group (each includes 2 mice). B) Differential expression analysis of transcriptional changes in response to LPS exposure and levosimendan treatment post‐LPS. |log2FC| ≥ 1; *P*_adj_ < 0.05. KEGG enrichment analysis of genes comparing LPS versus Sham C), and LPS + LEV versus LPS D). *P*_adj_ < 0.05. E) Heatmap depicting the differential expression of inflammation, immunity, and apoptosis‐related genes in the cortex of different groups. F) Representative Iba1 and GFAP immunostaining images in the cortex and hippocampus at 24 h after LPS injection in sham, LEV, LPS, and LPS + LEV groups (blue, DAPI; green, Iba1; red, GFAP; scale bar: 50 µm). *n* = 6–8/group. G–J) Quantification of the percentage of Iba1 and GFAP immunopositive areas relative to the total image area. K–N) Inflammatory mediator mRNA levels quantified by RT‐qPCR: CCL5, CXCL1, CXCL9, and CXCL10 at 24 h post‐LPS injection. *n* = 8/group. Statistical analysis: H,I,J,K) One‐way ANOVA; G,L,M,N) Kruskal‐Wallis test. Data are presented as means ± SEM. ^*^ *p* < 0.05, ^**^ *p* < 0.01 and ^***^ *p* < 0.001, as indicated.

Neuroinflammation serves as the central mechanism underlying brain injury in SAE, mediating or participating in the neurological damage caused by pathological alterations.^[^ ^53^, ^54^ ^]^ To explore the potential application value of improving the glymphatic system through CBF in SAE, we conducted transcriptomic analysis of the cortex at 24 h post‐LPS injection to investigate the effects of levosimendan treatment on the inflammatory microenvironment in the brain. Principal components analysis (PCA) revealed distinct clustering patterns among groups (**Figure** **6**A **)**. In model‐specific (LPS vs Sham) and intervention‐specific (LPS + LEV versus LPS) comparisons, we identified 1188 upregulated and 775 downregulated differentially expressed genes (DEGs), and 496 upregulated and 590 downregulated DEGs, respectively (Figure 6B). Kyoto Encyclopedia of Genes and Genomes (KEGG) pathway enrichment analysis revealed that LPS‐modulated genes were predominantly associated with pathways involved in inflammatory responses and innate immune regulation (*Z‐*score > 0, *P*_adj_ < 0.05; Top 10 pathways shown in Figure 6C), such as the TNF signaling pathway, IL‐17 signaling pathway, and NF‐kappa B signaling pathway. The suppression of these signaling pathways indicated that levosimendan treatment effectively maintained transcriptional homeostasis in the brain (Figure 6D). As shown in Figure 6E, LPS induced significant upregulation of a multitude of inflammatory‐related gene expressions in the cortex, including chemokines, inflammatory cytokines, and inflammasome components. The fluorescence intensity of CM‐injected CSF tracer in the MCA territory during the initial 30 min primarily indicates glymphatic influx, while measurements during the subsequent period correspond to the efflux process.^[^ ^33^, ^34^ ^]^ To analyze the correlation between glymphatic dysfunction and neuroinflammation, we measured the fluorescence intensity of BSA@IR‐780 at 60 min post CM‐injection (Figure S12A–D, Supporting Information), which reflects integration of the two processes, and collected cortical tissues post‐transcranial imaging. The expression of inflammatory factors, selected based on the primary differentially expressed genes revealed by RNA‐seq (Figure 6E), was quantified by qPCR. CCL5, CXCL1, CXCL9, and CXCL10 were significantly upregulated in the LPS‐treated group compared to sham controls (Figure S12E–K, Supporting Information). Moreover, the expression of these chemokines was positively correlated with in vivo imaging‐derived glymphatic metrics (intensity of CSF tracer BSA@IR‐780 in the MCA territory at 60 min post CM‐injection), suggesting a close relationship between glymphatic function and neuroinflammation in SAE (Figure S12L–R, Supporting Information). Levosimendan treatment inhibited the most upregulation of inflammation‐related genes induced by LPS (Figure 6E).

Our immunofluorescence staining showed that acute systemic LPS exposure increased the Iba1+ area in both the cortex and hippocampus, as well as the GFAP+ area in the cortex, suggesting hyperactivation of microglia and astrocytes. Levosimendan treatment, in contrast, suppressed the glial activation induced by LPS, in line with the anti‐inflammatory effects observed in RNA‐seq (Figure 6F–J). To determine whether levosimendan alleviates neuroinflammation by enhancing glymphatic function, we administered the glymphatic inhibitor TGN‐020 at 30 min after LPS induction. Notably, qPCR data indicated that TGN‐020 completely abolished levosimendan's suppressive effects on LPS‐induced elevation of pro‐inflammatory chemokines (CCL5, CXCL1, CXCL9, and CXCL10) (Figure 6K–N). Our findings establish glymphatic function as a critical mediator of levosimendan's anti‐neuroinflammatory efficacy in SAE.

### 2.7 Levosimendan Ameliorates LPS‐Induced Neurobehavioral Disorders

**Figure 7:**
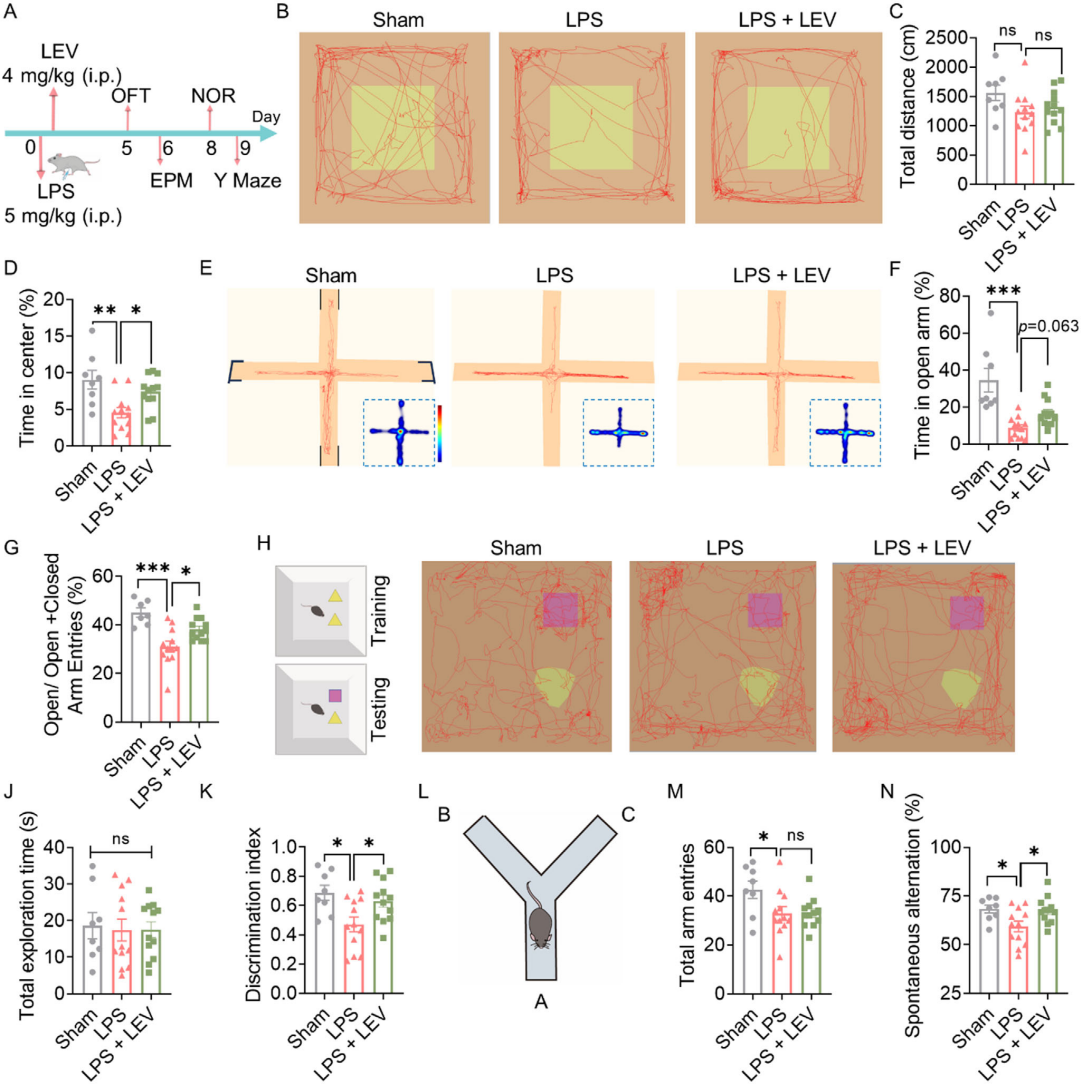
Levosimendan improves neurofunctional outcomes of LPS‐exposed mice. A) Schematic timeline for behavioral tests. Representative exploratory trajectories in OFT B) and EPM E) over 5 min. *n* = 8–12/group. Quantification of OFT parameters: total distance traveled C) and center time (%) D). EPM behavioral indices: F) % time in open arms; G) % open arm entries. H) Representative exploration paths from NOR. *n* = 8–12/group. Quantification of total object exploration time J) and discrimination index K) during NOR testing. L) Schematic of the Y‐maze apparatus. Y‐maze behavioral quantification: M) total arm entries; N) spontaneous alternation percentage. *n* = 8–12/group. Statistical analysis: C,D,G,J,K,M,N) One‐way ANOVA; F) Kruskal‐Wallis test. Data are presented as means ± SEM. ^*^ *p *< 0.05, ^**^ *p* < 0.01 and ^***^ *p* < 0.001, as indicated.

SAE is defined as a diffuse brain dysfunction, characterized by neurobehavioral disorders such as delirium and cognitive impairment.^[^ ^55^ ^]^ To explore the neuroprotective effects of levosimendan treatment at the brain functional level, we performed a series of behavioral tests to assess neuropsychiatric and cognitive functions in mice after resolution of overt sepsis symptoms induced by LPS. As illustrated in **Figure** **7**A, we sequentially performed the open field test (OFT), elevated plus maze (EPM) test, novel object recognition (NOR) test, and Y‐maze test on days 5, 6, 8, and 9 post‐LPS injection, respectively. The total distance traveled in the OFT showed that spontaneous locomotor activity had recovered in mice by day 5 after LPS injection (Figure 7B,C). However, both the OFT and subsequent EPM test revealed that LPS‐treated mice exhibited depressive‐like behaviors, showing significantly reduced center zone time in the open field (Figure 7B,D) and markedly decreased open arm exploration (fewer entries and shorter dwell time) in the EPM (Figure 7E–G), compared to mice in the sham group. Notably, levosimendan treatment significantly increased both center zone time in the open field (Figure 7B,D) and open arm entries in the EPM (Figure 7E,G) in LPS‐treated mice, demonstrating its ameliorative effect on LPS‐induced depressive‐like behaviors. Cognitive impairments persisted for more than 1 week post‐LPS, as evidenced by reduced novel object exploration time in the NOR test (Figure 7H–K) and decreased spontaneous alternation in Y‐maze test (Figure 7L–N); both were significantly improved by levosimendan treatment, demonstrating its efficacy against LPS‐induced cognitive dysfunction.

## 3 Discussion

Due to the limitations of traditional approaches for evaluating the glymphatic function, our understanding of the continuous and precise changes in the glymphatic system following systemic inflammation remains incomplete. A previous study used histological examination to assess the glymphatic influx within 3 h following the induction of systemic inflammation and concluded that LPS inhibits the inflow processes of the glymphatic circulation.^[^ ^22^ ^]^ However, we found that the systemic inflammation‐induced reduction in glymphatic influx was transient, which was followed by a more significant and prolonged increase in glymphatic influx until 72 h, as observed by NIR‐II in vivo imaging. The transient reduction in influx appears to be a temporary stress‐induced fluctuation in the glymphatic system when it is subjected to acute pathological stimuli. A similar phenomenon was observed in ischemic stroke model mice.^[^ ^21^, ^56^ ^]^ As the pathology progresses irreversibly, the glymphatic system ultimately undergoes pathological functional changes. Furthermore, to the influx, we also confirmed that the LPS challenge inhibits the glymphatic efflux process, which was previously unknown. One of the primary functions of the glymphatic efflux process is the clearance of metabolic waste products from the brain, especially for macromolecules, such as Aβ.^[^ ^57^, ^58^ ^]^ The impaired glymphatic clearance of Aβ that we observed provides additional evidence of the disrupted glymphatic efflux process following the induction of systemic inflammation, and indicates that glymphatic dysfunction mediates the accumulation of Aβ in SAE brains. It's known that the accumulation of Aβ is closely associated with cognitive impairment and dementia.^[^ ^59^, ^60^ ^]^

Notably, CSF dynamics cannot be fully equated with bulk water transport, as CSF movement involves complex, solute‐dependent mechanisms that extend beyond simple hydrodynamic flow. Structurally, the glymphatic system is a perivascular space formed by the astrocytic end‐feet and the outer wall of blood vessels.^[^ ^61^ ^]^ While AQP4 expression enables water to traverse the perivascular astrocytic endfeet barrier through both paracellular pathways (via inter‐endfeet spaces) and transcellular routes (through AQP4 water channels), classical tracers are restricted to paracellular transport via inter‐endfeet spaces due to the absence of specific transporters for them.^[^ ^8^, ^49^, ^62^ ^]^ The low molecular weight of water and the presence of AQP4 allow water to traverse the brain parenchyma at a significantly higher rate than classical tracers, as demonstrated by H_2_^17^O MRI experiments.^[^ ^63^ ^]^ Consequently, classical tracers, including our BSA@IR‐780, IR‐808, and IR‐808AC, inherently underestimate water flux. Fortunately, brain wet/dry weight measurements confirmed no edema in our model. Both classical tracers and water transport in the glymphatic system are AQP4‐dependent, as established in prior studies,^[^ ^9^, ^52^ ^]^ and further supported by our TGN‐020 inhibition experiments. AQP4 has been established as a critical therapeutic target for cerebral edema, where it mediates astrocytic cytotoxic edema in the acute phase but nevertheless alleviates vasogenic edema during the chronic phase.^[^ ^64^, ^65^, ^66^, ^67^, ^68^ ^]^ We observed that LPS treatment significantly reduced the polarized distribution of perivascular AQP4 at 24 h post‐induction, concomitant with downregulated AQP4 protein expression. The downregulated AQP4 protein at 24 h post‐LPS induction plausibly explains the lack of observable cerebral edema in this study. In fact, brain edema formation is not typically observed as a primary pathological manifestation in LPS‐induced experimental sepsis model.^[^ ^69^ ^]^ Beyond altered expression levels, disrupted AQP4 polarization may also involve rapid subcellular translocation, which is responsible for acute CNS edema in spinal cord injury and ischemic stroke.^[^ ^10^, ^11^, ^70^ ^]^ After endocytosis, AQP4's fate is determined within EEA1‐positive endosomes: either degradation in lysosomes or recycling back to the plasma membrane.^[^ ^71^, ^72^, ^73^ ^]^ In normal mice, the inhibition of glymphatic influx induced by either AQP4 knockout or AQP4 suppression (as in our experiments) appears contradictory to the LPS‐induced increase in influx that we observed.^[^ ^9^, ^52^ ^]^ These findings caution against interpreting AQP4 as a unidirectional regulator of flux, since its depolarization could alternatively indicate structural compromise of the astrocytic endfeet barrier. LPS treatment induces retraction of astrocytic endfeet, resulting in enlargement of the PVS.^[^ ^74^, ^75^ ^]^ A prior study in a colitis model similarly reported AQP4 depolarization concurrent with enhanced glymphatic influx, thereby corroborating our experimental observations.^[^ ^34^ ^]^

Compared to the brain, the heart is more vulnerable to sepsis due to its direct exposure to the cytokine storm induced by infection. It has been reported that in 98% of patients with severe sepsis, the heart failure biomarker N‐terminal pro‐brain natriuretic peptide (NT‐proBNP) is significantly elevated.^[^ ^76^ ^]^ Cardiac dysfunction and impaired cerebral autoregulation following sepsis collectively contribute to the reduction in CBF.^[^ ^77^, ^78^ ^]^ Levosimendan is a clinically approved and well‐established inotropic agent that enhances myocardial contractility by increasing the calcium sensitivity of contractile proteins through its binding to cardiac troponin C (cTnC).^[^ ^79^ ^]^ A previous study has demonstrated that levosimendan can improve cardiac function and reduce mortality in patients with sepsis.^[^ ^80^ ^]^ It is encouraging that levosimendan, by increasing CBF, effectively ameliorated LPS‐induced glymphatic dysfunction, improving both the influx and efflux processes, as well as enhancing Aβ clearance. The abolition of levosimendan's beneficial effect on glymphatic function by BCAS provides direct evidence that its mechanism involves enhancing cerebral perfusion. Another inotropic agent, digoxin, showed similar effects to levosimendan, while CBF showed a robust negative correlation with glymphatic influx. These findings strongly support the feasibility of improving glymphatic system function through cardiogenic enhancement of CBF. The blockade of levosimendan's effects by the AQP4 inhibitor TGN‐20 confirms that cerebral hypoperfusion promotes glymphatic dysfunction through AQP4. Levosimendan increased the expression and polarization of AQP4 along the cerebrovascular in LPS‐treated mice, indicating improvements in both the structure and function of the glymphatic system at the microscopic level. As an inotropic agent, levosimendan's pulsatility‐enhancing effects may operate through either indirect mediation via CBF augmentation or direct chronotropic action through increased heart rate. However, arterial pulsatility is well established as the driving force for glymphatic flux, exhibiting a positive correlation with glymphatic influx.^[^ ^44^ ^]^ This contradicts our observation of attenuated excessive influx, indicating that cerebral arterial pulsatility is not the primary cause of LPS‐induced glymphatic dysfunction. Wang et al. demonstrated that hypoperfusion leads to attenuated arterial pulsation.^[^ ^81^ ^]^ Consistent with this, our data show that restricting CBF suppresses the ameliorative effect of levosimendan on arterial pulsation. Together, these findings provide further evidence that arterial pulsation is regulated by CBF. Similarly, recent studies have identified slow vasomotion as an additional driver of glymphatic flux.^[^ ^82^, ^83^, ^84^ ^]^ The synergistic effects among vasomotion, arterial pulsatility, and hypoperfusion in LPS‐induced glymphatic dysfunction remain to be investigated.

Levosimendan inhibits LPS‐induced microglial activation in vitro, suggesting potential direct anti‐neuroinflammatory action.^[^ ^85^ ^]^ However, radiotracer evidence demonstrates BBB impermeability to levosimendan, precluding direct central activity after systemic delivery.^[^ ^86^ ^]^ Plaschke et al. demonstrated that intravenous levosimendan administered 24 h post‐LPS fails to attenuate neuroinflammation, consistent with its BBB impermeability.^[^ ^87^ ^]^ In contrast, our early intervention (5 min post‐LPS) alleviated neuroinflammation, revealing an early‐phase therapeutic window for glymphatic function rescue, with efficacy further validated by digoxin administration at 1 h post‐LPS induction. Disruption of the glymphatic system peaked at 24 h post‐LPS injection, by which time the system may have already undergone irreversible pathological changes. Moreover, using TGN‐020 as a pharmacological tool, we demonstrated that levosimendan suppresses LPS‐induced neuroinflammation by improving glymphatic system function. Neuroinflammation is a core pathological process in SAE, interacting with multiple pathological processes to form a vicious cycle that amplifies the damage to the nervous system.^[^ ^6^, ^88^ ^]^ Our results indicate that glymphatic dysfunction precedes neuroinflammation, acting as an upstream event. Additionally, the observed attenuation of neurobehavioral disorders provides substantial evidence supporting the therapeutic potential of levosimendan in SAE. However, the feedback effects of neuroinflammation on the glymphatic system remain unclear. Reactive gliosis following acute brain injury exhibits dual‐phase immunomodulation: during the acute phase, pro‐inflammatory astrocytes and microglia exacerbate neuroinflammation and compromise BBB integrity through cytokine/chemokine release, while anti‐inflammatory counterparts secrete neurotrophic factors to maintain metabolic homeostasis. In chronic stages, glial scar formation contains inflammatory spread, though simultaneously impedes axonal regeneration and synaptic remodeling.^[^ ^65^, ^89^ ^]^ Genetic ablation of AQP4 suppresses astrocyte activation.^[^ ^90^ ^]^ Meanwhile, reactive astrocytes exhibit disease‐stage‐dependent reprogramming of AQP4 expression profiles.^[^ ^91^ ^]^ Considering the pivotal role of AQP4 in glymphatic function, the regulation of astrogliosis‐mediated dynamic neuroinflammatory on glymphatic function warrants further investigation.

Most molecules are strictly restricted from crossing the BBB due to tight junctions and the extremely low levels of transcytosis in cerebrovascular endothelial cells.^[^ ^92^ ^]^ This implies that the efficiency of substance transport across the BBB is likely to be lower than that of the glymphatic system. Additionally, it should be noted that the development of SAE does not depend on the dysfunction of the BBB. In some patients with SAE, the BBB does not exhibit significant damage.^[^ ^93^ ^]^ The dose of LPS used in our study did not significantly increase BBB permeability, a finding confirmed in both our current and previous studies.^[^ ^88^ ^]^ Nevertheless, at this dose, LPS has already induced dysfunction of the glymphatic system. Based on these findings, we propose that alterations in glymphatic function may play a more extensive role in the development of SAE than previously appreciated.

Finally, it is important to acknowledge several methodological limitations in the present study. Beyond the advantages of direct hydrodynamic characterization, MRI can non‐invasively acquire whole‐brain 3D glymphatic dynamics, providing comprehensive visualization of pathway distribution (including PVS and meningeal lymphatic drainage routes), thereby avoiding the sampling bias inherent in 2D imaging techniques.^[^ ^52^, ^94^, ^95^ ^]^ This capability makes MRI particularly valuable for longitudinal studies and clinical translation of glymphatic system research. The glymphatic system was once challenged due to the use of inappropriate anesthetics.^[^ ^9^, ^96^ ^]^ Mice anesthetized with ketamine/xylazine or isoflurane/dexmedetomidine exhibit superior glymphatic influx compared to those administered Avertin, α‐chloralose, or isoflurane alone.^[^ ^97^ ^]^ AQP4 membrane anchoring requires stable interaction with the dystrophin‐associated protein complex in lipid rafts.^[^ ^98^ ^]^ Lipid‐soluble anesthetics (e.g., isoflurane) impair AQP4‐dependent glymphatic flux by reducing the central noradrenergic tone.^[^ ^99^ ^]^ The α_2_‐adrenergic receptor agonists like xylazine and dexmedetomidine may enhance glymphatic flow by modulating vasomotion via locus coeruleus norepinephrine inhibition.^[^ ^82^, ^100^ ^]^ Therefore, we employed the isoflurane /dexmedetomidine anesthetic protocol in this study to investigate the glymphatic transport of macromolecular tracers. Although Mestre et al. demonstrated parenchymal injection procedures impair glymphatic function, our work and others show invasive injections do not abolish glymphatic clearance, with intergroup differences remaining detectable.^[^ ^9^, ^101^, ^102^, ^103^ ^]^ Nevertheless, in light of Mestre H's findings, minimizing invasive procedures remains strongly recommended to optimize signal‐to‐noise ratios in glymphatic function assessments.

Overall, our results demonstrate that cerebral hypoperfusion is a key driver of systemic inflammation‐induced glymphatic dysfunction. Levosimendan, by targeting the glymphatic system, exhibits significant therapeutic potential for the treatment of SAE.

## 4 Experimental Section

### Animals

Male C57BL/6J mice, aged 8–10 weeks (22–25 g), purchased from Jilin Qianhe Model Biological Technology Co., Ltd., were housed in a temperature‐, humidity‐, and light‐controlled environment, with ad libitum access to food and water. This study was approved by the Ethics Committee of the First Hospital of Jilin University (approval number: 20210642 and JDYY20240329), and reported according to the ARRIVE guidelines. In this study, every effort was made to minimize both the number of animals used and the potential suffering they might endure.

### LPS and Drugs Administration

LPS Administration. The mice were randomly assigned to receive intraperitoneal injections of LPS (1 or 5 mg kg^−1^, Sigma‐Aldrich L2880) or an equivalent volume of sterile saline. At 5 min after LPS administration, the mice received intraperitoneal injections of levosimendan (4 mg kg^−1^, Solarbio, IL1140). Levosimendan was dissolved in dimethyl sulfoxide (DMSO) (Aladdin, D103276) and subsequently diluted in 60% saline (Solarbio, IN9000) and 35% PEG300 (Solarbio, IP9020). The control group received an equal volume of 5% DMSO, 60% saline, and 35% PEG300. Digoxin (0.15 mg kg^−1^; Selleckchem, S4290) was dissolved in DMSO and subsequently diluted in saline. Digoxin was administered intraperitoneally 1 h after LPS injection. Control groups received an equivalent volume of vehicle solution (1% DMSO in saline). Excluding animals that died following LPS injection or before the experiment, all animals were included. Mice were randomly assigned to each group. Researchers were blinded to the model and treatment groups during the experiment and quantification process.

### Cisterna Magna Injection and Transcranial Imaging

Mice were anesthetized under 4% isoflurane (RWD Life Science). During anesthesia, the mice breathed spontaneously, and their body temperature was maintained at ≈37°C using a heating pad. The mouse was secured on a stereotaxic frame, the skin was incised to expose the skull and CM, a needle connected to PE10 tubing was inserted into the CM, and 3 m tissue adhesive and dental cement were applied. Dexmedetomidine (Sigma‐Aldrich, SML0956) was administered via intraperitoneal injection 5 min before CM injection at a dosage of 0.2 mg kg^−1^, and isoflurane concentration was maintained at 1.5% during the surgery. The synthesis of BSA@IR‐780 has been detailed in our previous research^[31]^. High‐resolution mass spectrometry (HRMS) results confirmed that bovine serum albumin (BSA) could covalently bind to the IR‐780 molecule (Figure S13A,B, Supporting Information). BSA@‐IR780 (300 µm, 7 µL) was administered into the CM at a rate of 1 µL min^−1^ using a microinjection pump (Harvard Apparatus). NIR‐II fluorescence images were captured using a 2D InGaAs camera (Princeton Instruments, NIRvana‐640). All imaging experiments utilized an 808 nm laser with a power density of ≈0.065 W cm^−^^2^ passing through an 850 nm short‐pass filter. The luminescence emitted from BSA@IR‐780 was filtered using a combination of 1000 and 1100 nm long‐pass filters. Fluorescence images were collected at 5 min intervals starting immediately after injection.

After imaging, the brain was carefully removed, and fluorescence images were captured using NIRvana‐640. Subsequently, the brain was fixed in 4% paraformaldehyde (PFA) (LEAGENE, DF0135) overnight, sectioned into 100 µm coronal slices using a vibratome, and 12 brain slices were collected at 300 µm intervals, ranging from 1.6 mm anterior to 3.0 mm posterior to bregma. Fluorescence intensity was detected using the Leica Thunder and LAS X Widefield analysis systems.

At 30 min post‐intracisternal injection, mice underwent transcardial perfusion with phosphate‐buffered saline (PBS) (Solarbio, P1020). After cerebellar excision, brain tissues were homogenized in 600 µL PBS and centrifuged at 12 000 g for 10 min at 4°C. The supernatant was collected for quantification of the BSA@‐IR780 concentration using a standard curve.

### Imaging of Cervical Lymph Nodes

Mice were placed in the supine position after CM injection. dCLN were exposed, and the first image was captured 15 min after the start of CM injection, with subsequent images collected at 15 min intervals for 60 min. sCLN were exposed after neck hair was removed using a depilatory cream before imaging. Images were taken once every 10 min for 1 h, beginning 10 min after injection.

### Parenchymal Clearance Assay

The mice were secured in a stereotaxic frame, the skull was exposed, and holes were drilled at the following coordinates: anterior/posterior +0.6 mm from bregma; medial/lateral +2.0 mm. The molecular weight of IR‐808AC was directly identified using HRMS (Figure S13C, Supporting Information). The detailed synthesis procedure of IR‐808AC has been described in our previous study.^[^ ^45^ ^]^ 2 µL of IR@808AC (50 µM, 2 µL) was injected at a rate of 0.4 µL min^−1^ at a DV of −3.25 mm using a Hamilton syringe, and images of the head were taken by the NIRvana‐640 once every hour for 2 h, beginning at the 10 min after injection. After imaging, the brains were extracted and fixed in 4% paraformaldehyde overnight. Afterward, the brain was sectioned into 100 µm coronal slices, and 6 brain slices were collected at 600 µm intervals, ranging from 1.2 mm anterior to 1.8 mm posterior to bregma. The sections were analyzed using Leica Thunder and LAS X Widefield analysis systems. Following intraparenchymal injection of 2 µL IR‐808AC as described, mice were sacrificed at 10 min and 2 h post‐injection via transcardial perfusion with PBS. The whole‐brain was homogenized in 500 µL DMSO and quantified the fluorescence intensity using the NIR‐II camera. Clearance percentage was calculated as [(Mean fluorescence intensity at 0 h ‐ Fluorescence intensity at 2 h) / Mean fluorescence intensity at 0 h] × 100%.

Hair of the mouse hindlimb was removed using a depilatory cream before imaging. The coordinates for femoral vein imaging were consistent with those described above. IR‐808 was obtained from Beijing Zhongying Biotechnology Co., Ltd. Inject IR@808 (2.5 µM, 2 µL) at a rate of 0.4 µL min^−1^ at a DV of −3.25 mm using a Hamilton syringe. Images of the femoral vein were taken at 15, 45, and 65 min after injection under ISO/DEX anesthesia.

### Immunofluorescence Staining

The mice were anesthetized and perfused with 4°C PBS, and the brains were extracted. The brains were successively immersed in 4% PFA, 15% sucrose, and 30% sucrose. The brains were coronally sectioned at 20 µm using a cryostat. The sections were washed with PBS and subsequently permeabilized and blocked in PBS containing 10% goat serum (Solarbio, SL038), 5% BSA (Solarbio, A8010), and 1% Triton X‐100 (Solarbio, T8200). Sections were then incubated overnight at 4°C with primary antibodies: CD31(1:500; Merck, MAB1398Z), AQP4 (1:200; Proteintect, CL488‐16473), Iba1 (1:500; Abcam, AB178846) and GFAP (1:1000; Invitrogen, PA1‐10004). Following four 5 min PBS washes, sections were incubated for 1 h at room temperature with Alexa Fluor 488/594/647‐conjugated secondary antibodies (1:1000; Abcam), washed again with PBS, and mounted with DAPI‐containing antifade mounting medium (Solarbio, S2110).

After transcardial perfusion with PBS, skulls were fixed in 4% PFA at 4°C for 24 h. Meninges were microdissected in PBS under a stereomicroscope. Tissues were blocked in PBS containing 10% goat serum, 5% BSA, and 1% Triton X‐100 for 1 h at room temperature. Tissues were incubated with Alexa Fluor 488‐conjugated anti‐LYVE‐1 antibody (1:400; Invitrogen, 53‐0443‐82) at 4°C for 24 h. Following four 5 min washes in PBS, samples were mounted with anti‐fade reagent containing DAPI. For mice used to assess BBB permeability, 3‐kDa dextran‐TMR (ThermoFisher, D3328, 10 µg g^−1^) dissolved in saline was injected via the tail vein and allowed to circulate for 1 h before the aforementioned procedures. Following overnight incubation with the primary antibody CD31 CD31(1:500; Merck, MAB1398Z) at 4°C, Alexa Fluor‐488/594/647‐conjugated secondary antibodies (1:1000; Abcam) were employed to label IgG, dextran, and vascular endothelial cells, respectively. OVA‐647 (3 mg mL^−1^, 8 µL, ThermoFisher, O34784) was administered into the CM at a rate of 1 µL min^−1^ using a microinjection pump (Harvard Apparatus), under anesthesia with ISO and DEX. After 1 h, mice were perfused with PBS transcardially, followed by meningeal isolation and co‐staining with LYVE‐1 antibody as described above. AQP4, LEVY‐1, and OVA‐647 were imaged an Olympus FV3000 confocal laser scanning microscope. Iba1, GFAP, dextran, and IgG were imaged with a fluorescence microscope (ZEISS, Axio Imager Z2, ApoTome.2 optical sectioning). ImageJ was employed to measure fluorescence density and area. The ratios of AQP4, dextran, and IgG fluorescence intensity relative to CD31‐positive area were calculated.

To quantify AQP4 polarization, 30 µm segments centered on blood vessels were analyzed using the line‐plot tool in ImageJ. Peak fluorescence intensity was measured within the perivascular 15 µm territory, with baseline fluorescence intensity recorded elsewhere. The baseline‐corrected peak fluorescence (peak minus baseline) was normalized to the adjusted peak intensity.

### Brain Edema

Following deep anesthesia, mouse brains were harvested and immediately weighed to obtain wet weight. Tissues were then desiccated in an 80°C oven for 72 h before dry weight measurement. The percentage of brain water content was calculated as: Brain water content (%) = [(Wet weight ‐ Dry weight) / Wet weight] × 100%.

### BCAS Model

Mice were anesthetized with ISO and subjected to BCAS at 3 h after LPS expose. The BCAS model was established by coiling microcoils (inner diameter: 0.18 mm, pitch: 0.5 mm, length: 2.5 mm; Beijing Xinong Technology Co., Ltd.), specifically designed for mice, around both common carotid arteries. For sham controls, the surgical site was exposed, and the common carotid arteries (CCA) were gently touched with forceps without coil insertion, followed by wound closure with tissue adhesive. Glymphatic function was evaluated at 24 h after LPS injection.

### Laser Speckle

Laser speckle flowmetry was used to measure CBF across different groups at baseline, before LPS injection, and at 1, 3, 24, and 72 h post‐injection. In addition, acute alterations were monitored in CBF following microcoil‐induced BCAS, as well as CBF changes at 24 h after BCAS. Mice were sedated with 4% isoflurane and maintained sedation with 1.5% isoflurane. A heating pad was used to stabilize the body temperature. Hair was removed, and the scalp was incised to expose the skull. CBF was assessed using a laser speckle blood‐flow imager (Sinowell, China).

### In Vivo Two‐Photon Fluorescence Imaging

Arterial pulsatility was evaluated using Olympus in vivo two‐photon microscopy. The cerebral vessels were labeled by venous injection of FITC‐2000 kDa. A Mai Tai laser was tuned to 780 nm to excite FITC‐2000 kDa. Emission was filtered at 495‐540 nm. To measure arterial pulsatility, X‐T line scans were used to measure the change in diameter of the surface arterial vessel versus time through a transparent skull window. Vessel diameters were quantified from X–T plots using cellSens software (Olympus). Vasomotion was quantified by calculating the absolute area under the diameter‐time curve over the 2000 ms period (units: µm × ms). The effects of LPS and levosimendan on cortical arterial pulsations were examined at 3 h post‐LPS administration. Additionally, BCAS was induced at 3 h after LPS injection, and its impact on arterial pulsations was further investigated following a 2 h recovery period.

### Physiological Indicator Assessment

The heart rate and respiratory rate were measured 24 h after LPS injection. In mice anesthetized with isoflurane, 1 mL syringe needles were inserted subcutaneously into the four limbs and then connected to electrodes. The body temperature of these mice was maintained at ≈37°C. A multifunctional physiological monitoring system for small animals and sftrepaly software were employed to collect and analyze electrocardiogram signals. Continuous monitoring was performed for 5 min, and the average value was calculated to represent the heart rate of the mice. The mice were lying prone with a respiratory transducer secured to their abdomen. The respiratory rate was recorded for 5 min, and the average value was calculated. Mice were anesthetized with tribromoethanol before and at 3 and 24 h after LPS injection. Cardiac ultrasound was performed using a Logiq e system (GE Healthcare), and changes in left ventricular ejection fraction (LVEF) and fractional shortening (FS) were calculated. Blood pressure and heart rate were measured in conscious mice using the tail‐cuff plethysmography method with a Medlab system (Nanjing Kalwen Co., Ltd, China). Values were averaged over three consecutive measurement cycles.

### Hippocampal Injection

The mice were anesthetized and placed on a stereotaxic frame. Head hair was cut, the skull was exposed, and a small hole was drilled at the coordinates (anterior/posterior −2 mm from the bregma; medial/lateral +1.5 mm). 1 µL of FITC‐labeled Aβ (1–42) (PLLABS, P2000022‐FITC), dissolved in artificial CSF (Solarbio, H7701) at a concentration of 0.5 mg mL^−1^ was injected at a rate of 0.2 µL min^−1^ at a DV of −2 mm using a Hamilton syringe. After 1 h, the brains were removed and fixed overnight in 4% PFA. The brains were sectioned coronally into 100 µm‐thick slices using a vibratome, and the sections were subsequently examined with a single‐photon confocal microscope (Olympus FV3000). The AQP4‐specific inhibitor TGN‐020 (250 mg kg^−1^, MCE, HY‐W008574), dissolved in 5% DMSO and 20% sulfobutylether‐β‐cyclodextrin (MCE, HY‐17031) was administered via intraperitoneal injection 15 min before stereotaxic hippocampal injection.

### Quantitative Real‐Time PCR (q‐PCR)

TGN‐020 (250 mg kg^−1^, MCE, HY‐W008574) was administered intraperitoneally 30 min after LPS injection. At 24 h post‐LPS injection, mice were transcardially perfused with ice‐cold PBS, followed by harvesting of brain tissues. Total RNA was extracted from the homogenized samples using the FastPure Cell/Tissue Total RNA Isolation Kit V2 (Vazyme, Cat# RC112‐01). Reverse transcription was performed using HiScript II Q Select RT SuperMix (Vazyme, Cat# R233‐01). qPCR was conducted using the ChamQ SYBR qPCR Master Mix (Vazyme, Cat# Q321‐02). The sequences of all primers used in this experiment are provided in Table S1 (Supporting Information).

### RNA Sequencing

Following anesthesia, the mice underwent cardiac perfusion with 4°C PBS. The cerebral cortex was then dissected on ice, immediately snap‐frozen in liquid nitrogen, and stored at −80°C for subsequent analysis. Tissue samples were obtained from four experimental groups: sham, sham+LEV, LPS, and LPS+LEV, with six mice per group. For RNA sequencing analysis, samples from the same group were randomly paired and pooled before being sent to Baihao Biotechnology Co., Ltd. in Shenyang for further processing. PCA was conducted using the prcomp function. Differential expression analysis was performed with the DESeq2 package, with significantly DEGs identified using the threshold criteria: |log_2_FC| ≥ 1 and *P*_adj_ < 0.05. KEGG pathway enrichment analysis were conducted using the clusterProfiler package. Data visualization was generated with the ggplot2, pheatmap, and GOplot packages. All analyses were performed in *R* statistical computing environment (version 4.4.0).

### Western Blotting

Mice underwent cardiac perfusion with ice‐cold PBS at 24 h post LPS injection. The cerebral cortex was immediately dissected on ice and homogenized in RIPA lysis buffer (Solarbio, R0020) supplemented with protease inhibitor cocktail (Solarbio, P6730). Proteins (50 µg per sample) were separated by 4%–12% SDS‐polyacrylamide gels and electrophoretically transferred to polyvinylidene difluoride membranes. Membranes were blocked with 5% non‐fat dry milk (Solarbio, D8340) in TBST (Solarbio, T1082) for 1 h at room temperature, followed by overnight incubation at 4°C with primary antibodies against AQP4 (rabbit polyclonal, 1:3000; Proteintech, 16473‐1‐AP) and β‐actin (rabbit monoclonal, 1:5000; Proteintech, 20536‐1‐AP). Following four sequential 5 min TBST washes, membranes were incubated with HRP‐conjugated goat anti‐rabbit IgG (1:10000; Proteintech, SA00001‐2) for 1 h at room temperature, followed by four additional 5 min TBST washes. Protein bands were detected with HRP chemiluminescent substrate (Merck, WBKLS0500) and imaged using FUSION SOL06S.EDGE. Band intensities were quantified by ImageJ (version 1.8.0) and normalized to β‐actin on the same membrane.

### Evans Blue Leakage Assay

Mice received 2% Evans Blue dye (Solarbio, E8010; 4 mL kg^−1^) via tail vein injection 4 h before sacrifice. As positive controls, some mice were subjected to pMCAO to induce BBB leakage. Under deep anesthesia, transcardial perfusion with PBS was performed to clear intravascular dye. The brains were imaged dorsally and ventrally, and sectioned coronally into 2 mm slices. Tissue sections were photographed, homogenized in 50% trichloroacetic acid, and centrifuged at 12000 g for 15 min (4°C). Evans Blue concentration in the supernatants was measured at 620 nm. Quantification was calculated against a standard curve and expressed as micrograms per gram of brain tissue (µg/g tissue).

### Behavioral Experiments‐OFT

The OFT was conducted in a square arena (40 × 40 × 35 cm, L × W × H). Each mouse was gently placed in the central zone facing a consistent orientation and allowed to explore freely for 5 min. Behavioral parameters (total distance traveled and center zone duration) were automatically tracked using the Noldus EthoVision XT 16. The arena was thoroughly cleaned with 75% ethanol between trials.

### EPM Test

The EPM consisted of two open arms (30 × 5 cm) and two enclosed arms (30 × 5 cm) connected by a central platform (5 × 5 cm). Mice were placed on the central platform facing an open arm and allowed to explore freely for 5 min. The percentage of time spent in open arms and the percentage of open‐arm entries were automatically tracked using the Noldus EthoVision XT 16. The apparatus was thoroughly cleaned with 75% ethanol between trials to eliminate olfactory cues.

### NOR Test

The NOR test was conducted using the same square arena as in the OFT. During the training phase, two identical triangular objects were placed in the arena, and mice were allowed to freely explore for 5 min per day. On day 3 (test phase), one familiar triangular object was replaced with a cube‐shaped object. Mouse exploration behavior was tracked using Noldus EthoVision XT 16. Exploration time directed toward the novel and familiar objects was measured. Total exploration time (novel + familiar objects) and novel object exploration ratio were calculated.

### Y Maze Test

The Y‐maze apparatus comprised three symmetrical arms (35 cm L × 10 cm W × 15 cm H) extending at 120° angles. Mice were placed at the distal end of one arm and permitted free exploration for 8 min. The sequence of arm entries was recorded for subsequent calculation of spontaneous alternation. A successful alternation was defined as three consecutive entries into different arms (e.g., ABC, BCA). The spontaneous alternation percentage was calculated as: Spontaneous alternation (%) = [Number of successful alternations/ (Total arm entries ‐ 2)] × 100, as described previously.^[^ ^104^ ^]^

### Statistical Analysis

Fluorescence image processing and quantification were performed using the ImageJ software (version 1.8.0). The protocol for processing images of the NIR‐II tracer influx was as follows: Three territories of interest (ROIs) were manually outlined in the left MCA territory, right MCA territory, and around the OB. The average intensity for each ROI was computed. The images were subsequently converted to RGB format and exported. The method for calculating the distribution of tracers within the cervical lymph nodes, blood vessels, bones, liver, and other organs was similar to that used for glymphatic inflow quantification. The ROI was manually outlined following the contours depicted in the brightest images. Quantifying tracer distribution in coronal brain slices involved manually tracing the contours of each slice and calculating the mean intensity within them. The total intensity of all slices was summed and divided by the number of slices to determine the intensity value for each mouse. Normalization was performed on data from quantitative analyses of AQP4 polarization, protein expression, inflammatory factors, cardiac function, and CBF. Data analysis was performed using SPSS Statistics 26 and GraphPad Prism8. The Shapiro–Wilk test was performed to assess the normality of the data. For normally distributed data, intergroup comparisons utilized Student's *t*‐test (two groups; *n* = 4–9/group) or one‐way ANOVA (multiple comparisons; *n* = 4–12/group). Repeated‐measures data were analyzed via two‐way repeated‐measures ANOVA (*n* = 4–9/group). Pearson correlation assessed variable associations (*n* = 6/group). For non‐normally distributed data, Mann‐Whitney *U* tests(*n* = 6–8/group) compared two groups while Kruskal‐Wallis tests (*n* = 4–12/group) handled multiple comparisons. Variable associations were evaluated using Spearman correlation analysis (*n* = 13/group). All scientific illustrations were prepared using Adobe Illustrator 2021. All values are presented as mean ± SEM. *p *< 0.05 was considered statistically significant.

## Conflict of Interest

The authors declare no conflict of interest.

## Author Contributions

R.Z., B.S., and P.W. contributed equally to this work. Z.G., Y.Y., and S.Z. conceived and designed the study. R.Z., B.S., and P.W. performed the research experiments. B.S. provided the NIR‐II fluorescence probes. R.Z. and P.W. analyzed the experimental data and wrote the manuscript. Q. H. and K. Z. checked up the data. Z. G., Y.S., and J.L. revised the manuscript and provided experimental advice. All the authors have read and approved the final version of the manuscript.

## Supporting information



Supporting Information

## Data Availability

The data that support the findings of this study are available from the corresponding author upon reasonable request.
