# Supplementary material for: Potentiating Cerebral Perfusion Normalizes Glymphatic Dynamics in Systemic Inflammation
Source: Adv Sci (Weinh). 2025 Oct 5;12(47):e03576. doi: 10.1002/advs.202503576 (PMC12713049; doi:10.1002/advs.202503576)
Supplement: Supplementary file 1 — Supporting Information [file ADVS-12-e03576-s001.pdf]

Supporting Information

Potentiating Cerebral Perfusion Normalizes Glymphatic Dynamics in Systemic Inflammation

Ruoyu Zhao†*, Bin Sun*†*, Pengju Wei*†*, Yingying Sun, Qianyan He, Kejia Zhang, Jun Lu, Shoujun Zhu *, Yi Yang*, Zhenni Guo*.*

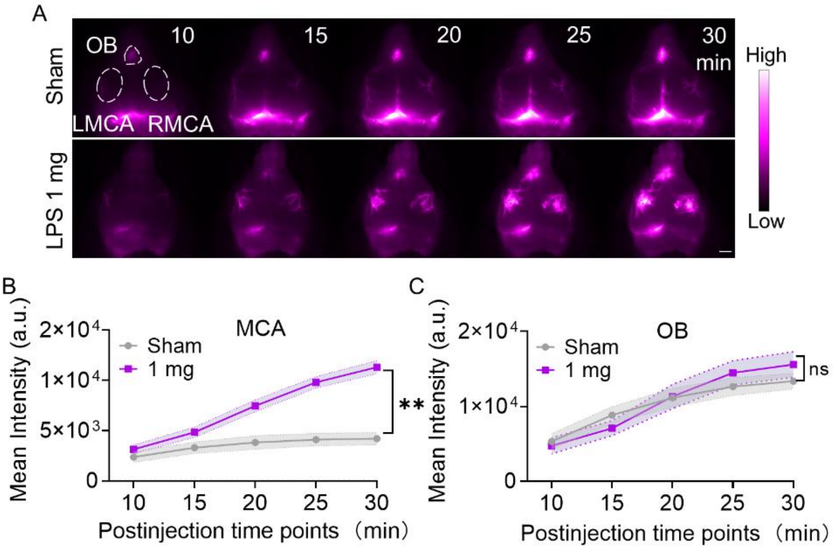

Figure S1. 1 mg/kg LPS increases glymphatic influx. (A) Representative in vivo NIR-II images showing CM- injection BAS@-IR780 (CSF tracer) influx at 24 h after a 1 mg/kg LPS i.p. injection for 30 minutes, with a 5-min interval. The dotted white line delineates the area designated for quantification. Sham, n = 4; LPS, n = 5. White scale bar: 2 mm. (B, C) Mean pixel intensity in arbitrary units (A.U.) around MCA/OB for 30 min post-CM injection, analazed by two-way repeated-measures ANOVA. Data are presented as means ± SEM. ** $p < 0.01$.

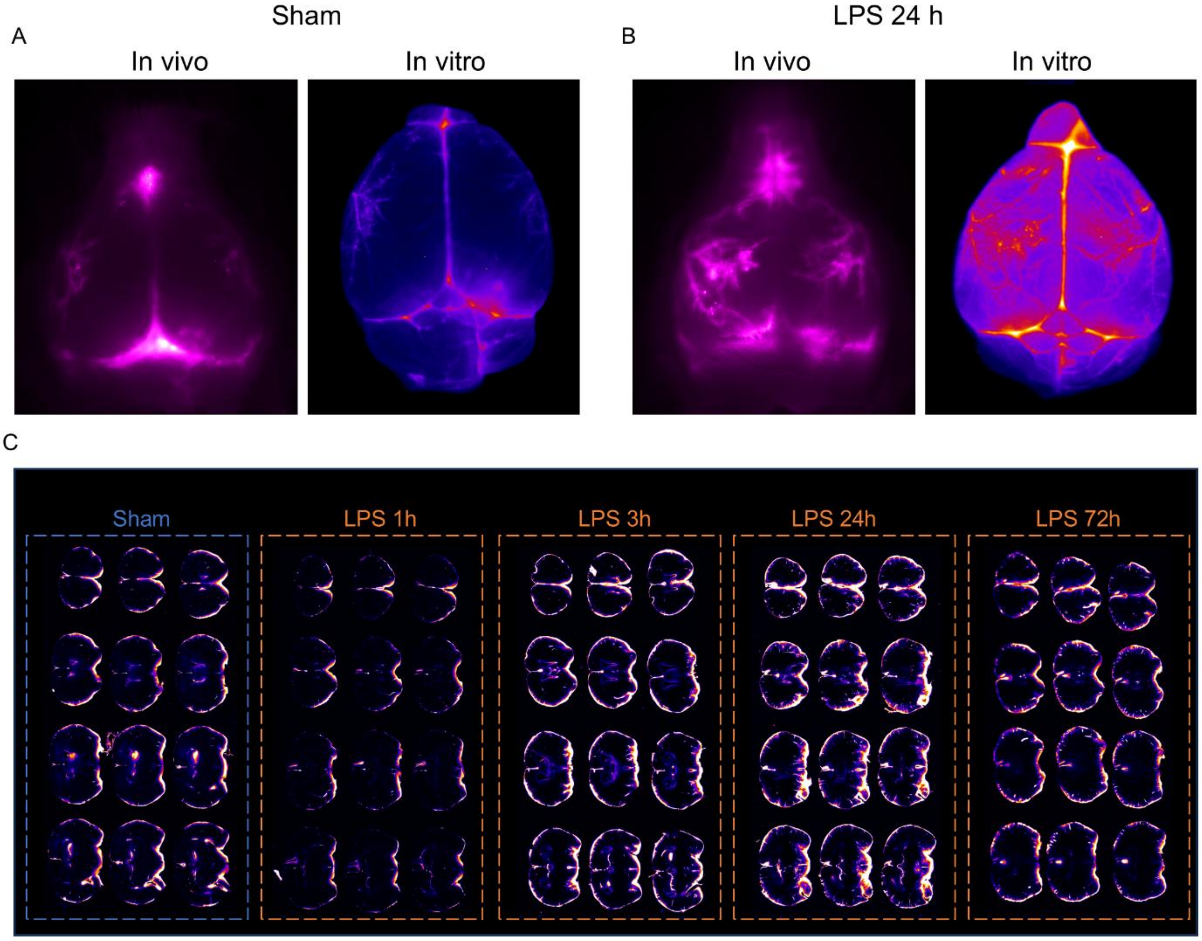

Figure S2. LPS alters NIR-II tracer distribution in the brain. Representative images in vivo, in vitro and dorsal brain distribution of BSA@IR-780 at 30 min post CM-injection: (A) Sham, (B) LPS. (C) Representative coronal sections depicting BAS@-IR780 CSF tracer influx (AP from +1.6 mm to -3.0 mm).

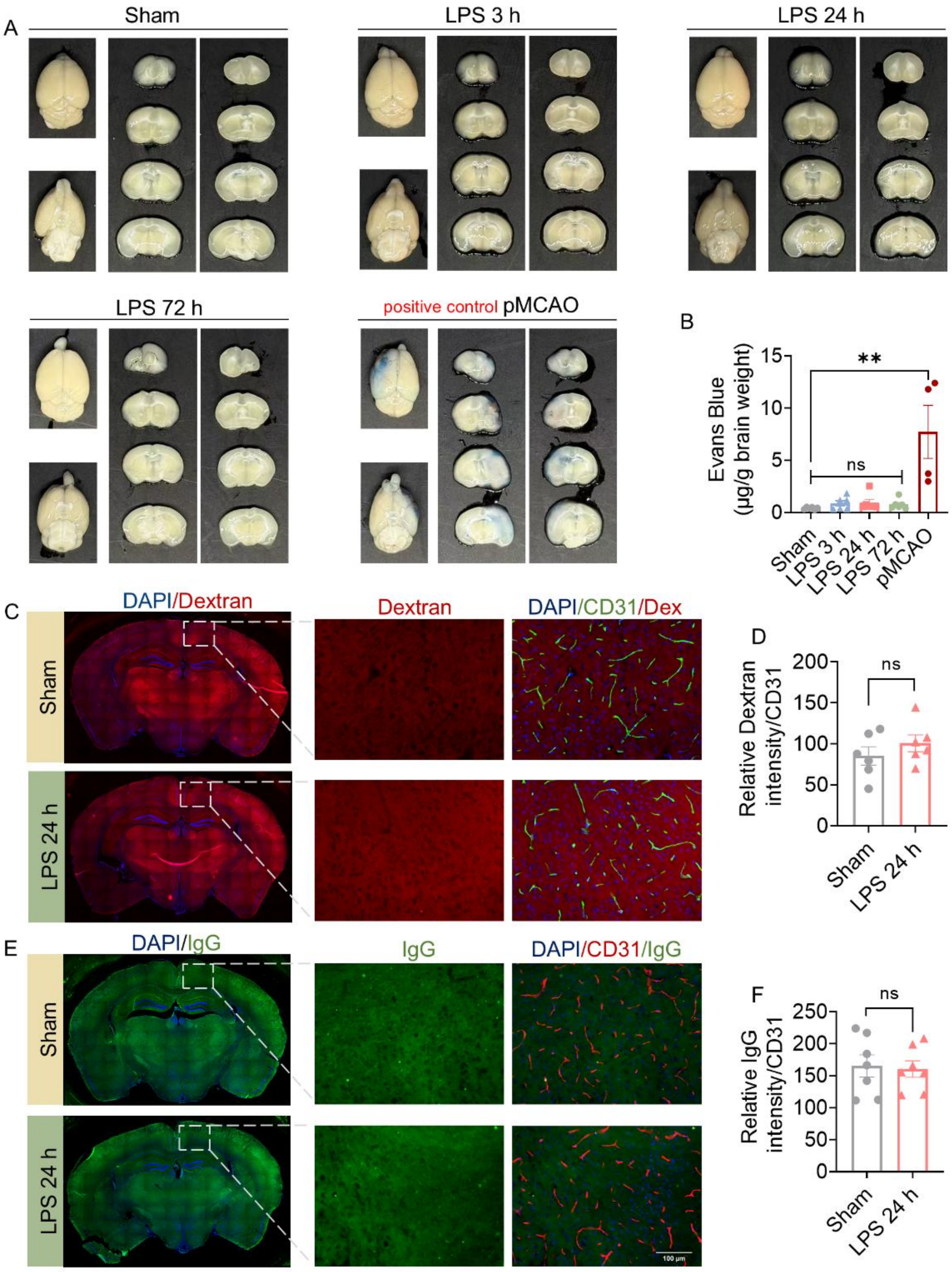

Figure S3. LPS does not increase BBB permeability. Representative images (A) and quantification (B) of Evans Blue leakage in brains of LPS-treated mice (3 h, 24 h, 72 h post-injection) and pMCAO model mice. LPS (n = 6/group); pMCAO (n = 4). (C, E) Representative images of Dextran and IgG leakage on 24 h after 5 mg/kg LPS injection (blue, DAPI; green, IgG; red, Dextran; scale bar: 100 μm, n = 6/group). (D, F) Quantification of Dextran and IgG leakage in the cortex (mean intensity of Dextran or IgG / CD31 area). Statistical analysis: (B) Kruskal-Wallis test; (D, F) Student's *t*-test. Data are presented as means ± SEM. ** $p < 0.01$.

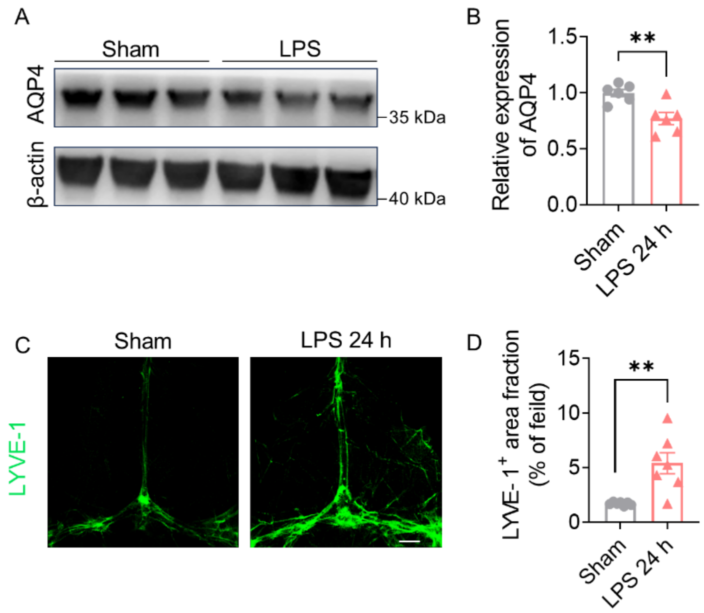

Figure S4. LPS reduces cortical AQP4 expression and promotes dorsal cranial meningeal lymphangiogenesis. (A) Representative AQP4 Western blot images and (B) statistical plots 24 h post-LPS. n = 6/group. (C) Representative immunofluorescence images of LYVE-1$^+$ lymphatic vessels in the cranial meninges. (D) Quantitative analysis of the LYVE-1$^+$vessel area. Scale bar: 1 mm. n = 7/group. Statistical analysis: (B, D) Student's *t*-test. Data are presented as means ± SEM. ** $p < 0.01$.

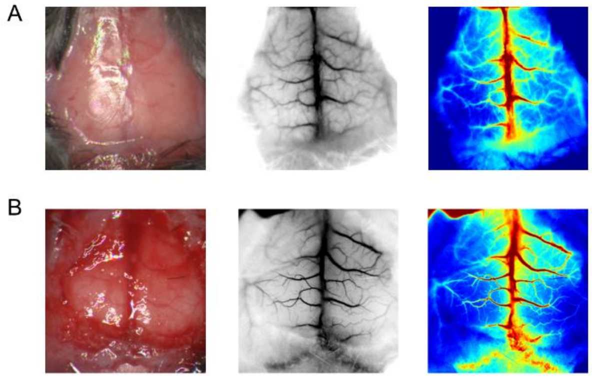

Figure S5. Laser-speckle flowmetry of CBF. Representative CBF images in mice: (A) intact skull, (B) cranial window.

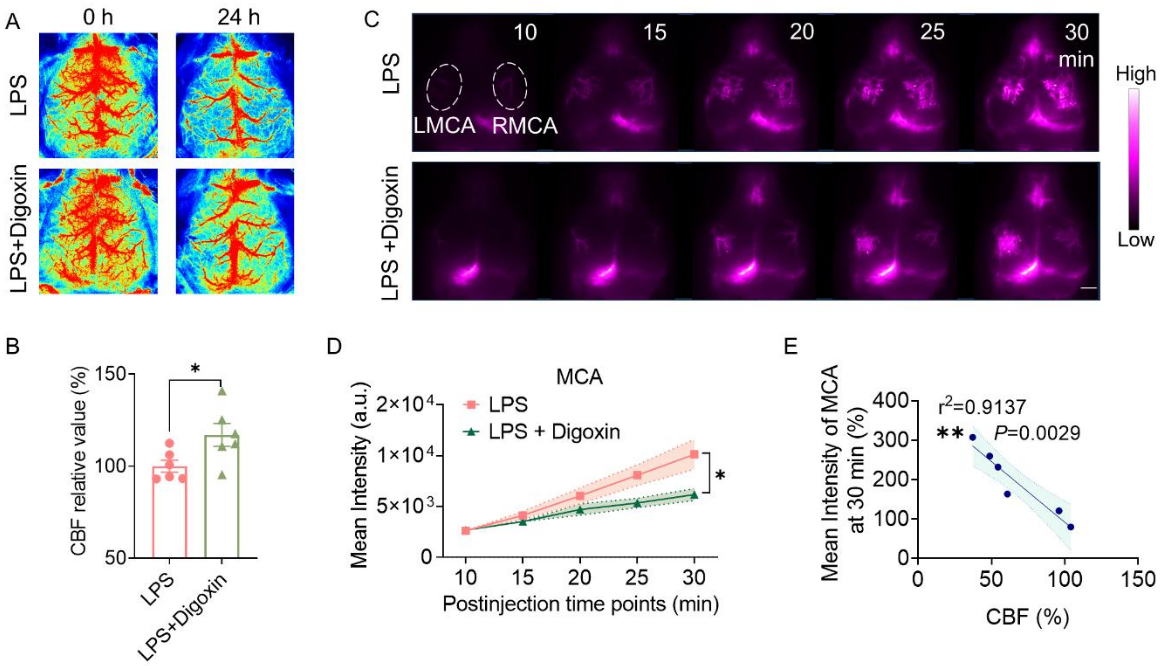

Figure S6. Digoxin improves CBF and suppresses glymphatic influx. (A) Representative images of CBF by laser speckle in LPS and LPS + Digoxin groups 24 h after LPS injection. n = 6/group. (B) Relative CBF in LPS + Digoxin vs. LPS groups at 24 h post-injection (%) (C) Representative in vivo images of BSA@IR-780 distribution. (n = 6/group) White scale bar: 2 mm. (D) Quantification of BSA@IR-780 distribution around MCA. (E) Correlation between relative CBF (%) and relative peri-MCA fluorescence intensity (%) in vivo 30 min after CM injection. n = 6/group. Statistical analysis: (B) Student's *t*-test; (D) Two-way repeated-measures ANOVA; (E)Pearson correlation. Data are presented as means ± SEM. * $p < 0.05$, ** $p < 0.01$.

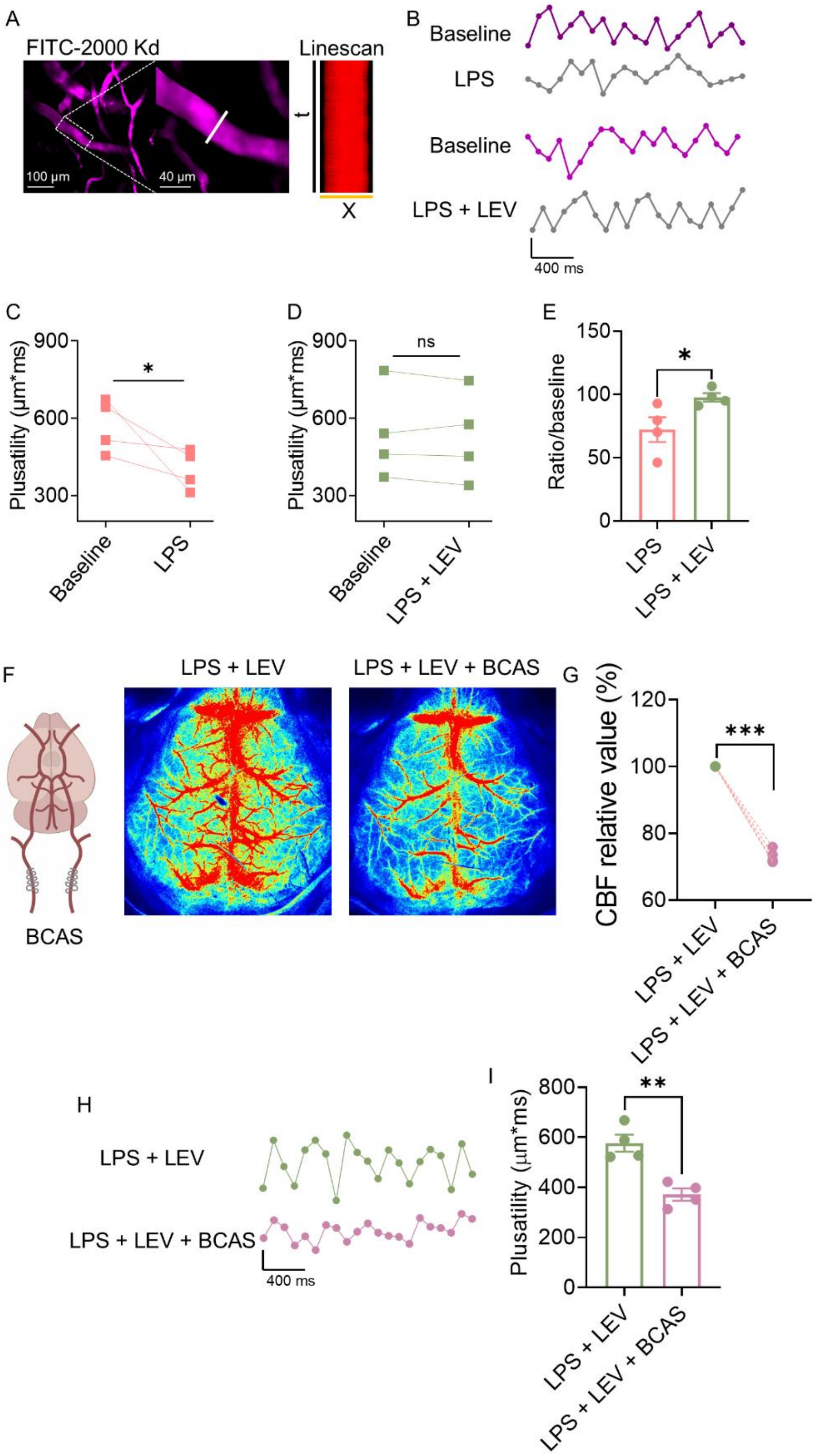

Figure S7. BCAS abolishes levosimendan-induced improvement in arterial pulsatility. (A) Representative imaging of surface vessels using two-photo microscopy after intravenous injection of FITC-2000 kDa. The X-T line scans were used to measurement of artery pulsatility. (B) Time-series of diameter of surface artery vessels. (C, D) The change in arterial pulsatility after LPS and LEV adiminstration. (D) The change in arterial pulsatility after LPS exposure with levosimendan treatment. (E) Percentage change in arterial pulsatility following LPS and

LPS+LEV treatment. n = 4/group. Images (F) and quantitative analysis (G) of CBF changes following microcoil-induced BCAS. n = 4. CBF measurements were obtained before and after microcoil placement, with a 5-8 min interval for the BCAS procedure, serving as its own control. (H) Time-series of artery diameter (H) and quantification (I) in LPS + LEV and LPS + LEV + BCAS groups. n = 4/group. Statistical analysis: (C, D, E, G, I) Student's *t*-test. Data are presented as means ± SEM. * $p < 0.05$. Data are presented as means ± SEM. * $p < 0.05$, ** $p < 0.01$ and *** $p < 0.001$.

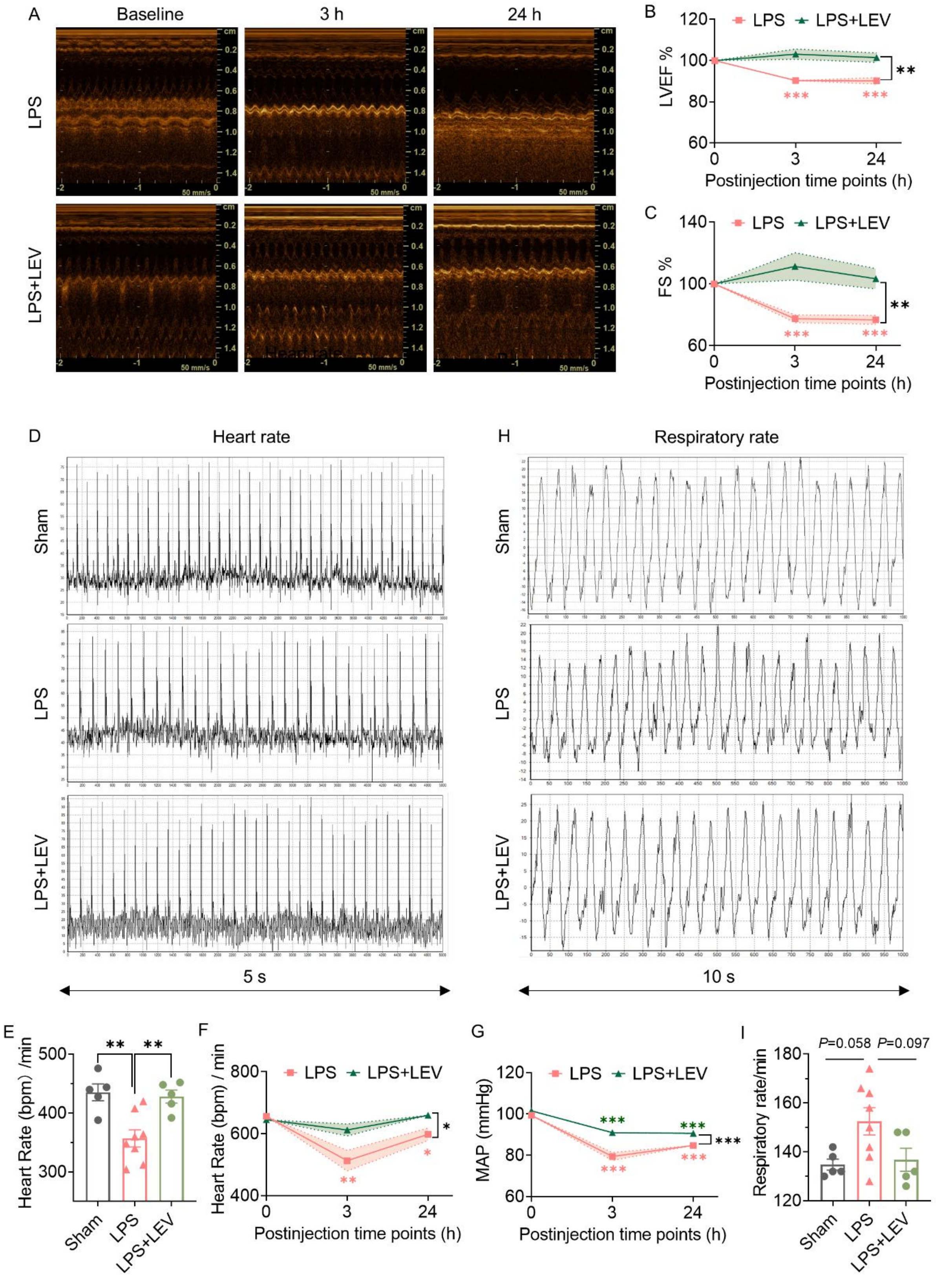

Figure S8. Levosimendan normalizes LPS-induced abnormalities in physiological parameters. (A) Representative images of echocardiography in LPS, LPS + LEV groups. n = 5-6/group. (B, C) Temporal changes in LVEF and FS relative to baseline in the LPS and LPS+LEV groups. Red and green asterisks (*) denote statistically significant changes from baseline in the LPS

and LPS+LEV groups, respectively.Heart rate images (D) and quantification (E) in isoflurane-anesthetized mice. n = 5-8/group. Heart rate (F) and blood pressure (G) in awake mice. n = 5-6/group. Respiratory rate images (H) and quantification (I) in isoflurane-anesthetized mice. n = 5-8/group. Statistical analysis: (B, C, F, G) Two-way repeated-measures ANOVA; (E, I) One-way ANOVA; Data are presented as means ± SEM. * $p < 0.05$, ** $p < 0.01$ and *** $p < 0.001$.

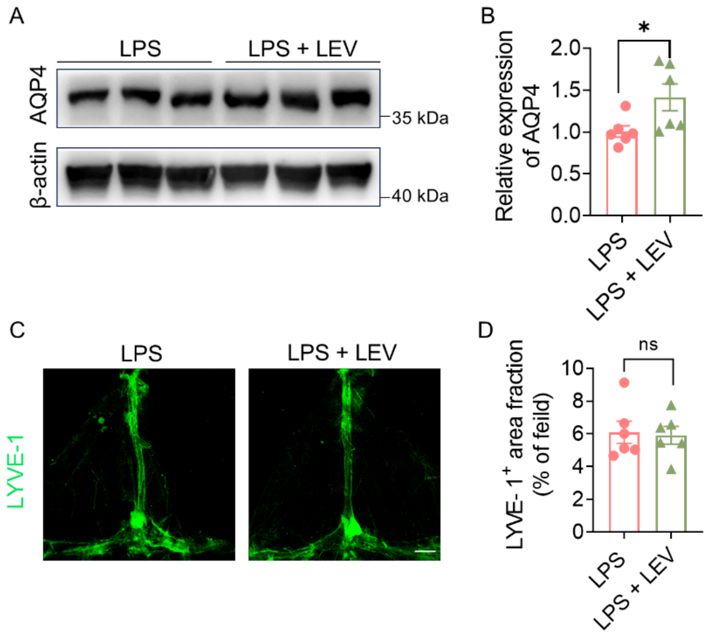

Figure S9. Levosimendan attenuates LPS-induced downregulation of cortical AQP4 expression. (A) Representative AQP4 Western blot images and (B) Quantitative data 24 h post-LPS. n = 6/group. (C) Representative immunofluorescence images of LYVE-1⁺ meningeal lymphatic vessels. Scale bar: 1 mm (D) Quantification of LYVE-1⁺ vessel area. n = 6/group. Statistical analysis: (B, D) Student's *t*-test. Data are presented as means ± SEM. ** $p < 0.01$.

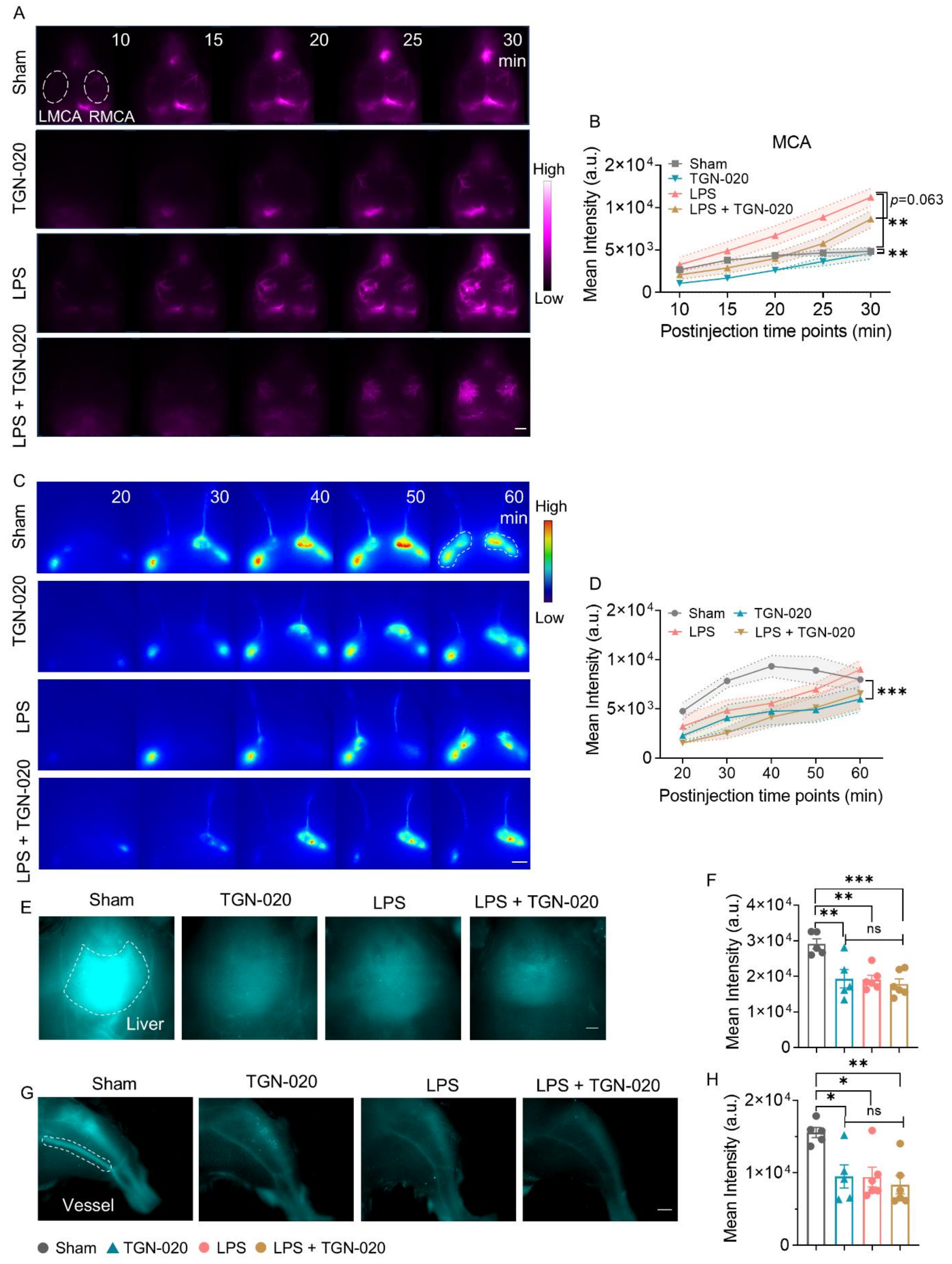

Figure S10. TGN-020 impairs glymphatic function in controls but not LPS-treated mice. Representative in vivo images (A) and quantitative analysis (B) of BSA@IR-780 influx within 30 min post CM-injection (n = 5-6/group). White scale bar: 2 mm. Representative images (C) and quantitative analysis (D) of BSA@IR-780 distribution on sCLNs within 1 h post-CM

injection. (n = 5-6/group). White scale bar: 2 mm. Representative NIR-II images of liver (E) and vessels (G) after monitoring sCLNs. n = 5–6/group. White scale bar: 2 mm. Quantification of fluorescence intensity in liver (F) and vessels (H). Statistical analysis: (B, D) Two-way repeated-measures ANOVA; (F) One-way ANOVA; (H) Kruskal-Wallis test. Data are presented as means ± SEM. * $p < 0.05$, ** $p < 0.01$, *** $p < 0.001$.

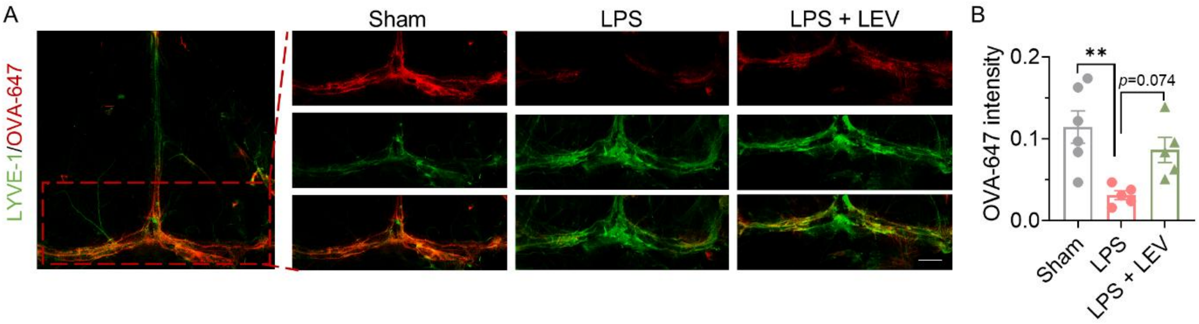

Figure S11. Levosimendan rescues meningeal lymphatic drainage impaired by LPS. (A) LYVE-1 immunostaining and OVA-647 distribution in meningeal. (green, LYVE-1; red, OVA-647; scale bars: 1 mm). n = 5-6/group. (B) Quantification of OVA-647 intensity. Statistical analysis: (B) One-way ANOVA. Data are presented as means ± SEM. ** $p < 0.01$.

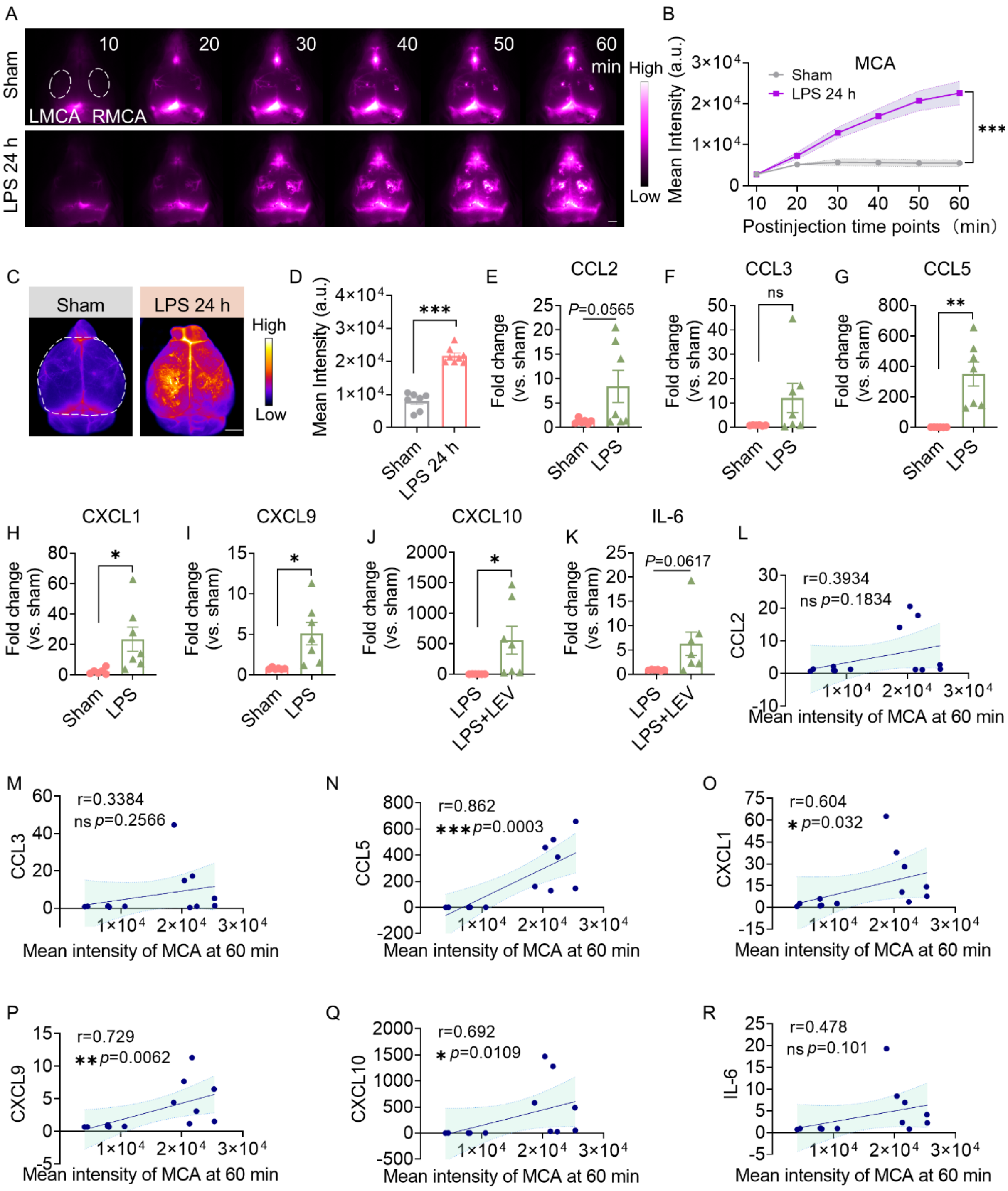

Figure S12. Glymphatic system disorder is positively correlated with LPS-induced neuroinflammation. (A) Representative in vivo images showing the distribution of the perivascular CSF tracer in sham and LPS groups within 1 hour after CM injection (n = 5/group). White scale bar: 2 mm. Assessment of fluorescence intensity around the MCA (B). (C) Representative images of BSA@IR-780 distribution in the dorsal cortex of sham and LPS-treated mice 1 h post-CM injection. n = 7-8/group. White scale bar: 2 mm. (D) Quantitative analysis of fluorescence intensity on the dorsal surface of the brain. (E-K) Quantitative RT-

PCR analysis of inflammatory mediator mRNA expression in the cerebral cortex 24 h after LPS exposure. n = 6-7/group (L-R) Correlation between inflammatory mediator mRNA expression and peri-MCA fluorescence intensity in vivo 60 min after CM injection. n = 13. Statistical analysis: (B) Two-way repeated-measures ANOVA; (D, G-K) Student's t-test; (E, F) Mann-Whitney *U* test; (L-R) Pearson correlation. Data are presented as means ± SEM. * $p < 0.05$, ** $p < 0.01$ and *** $p < 0.001$.

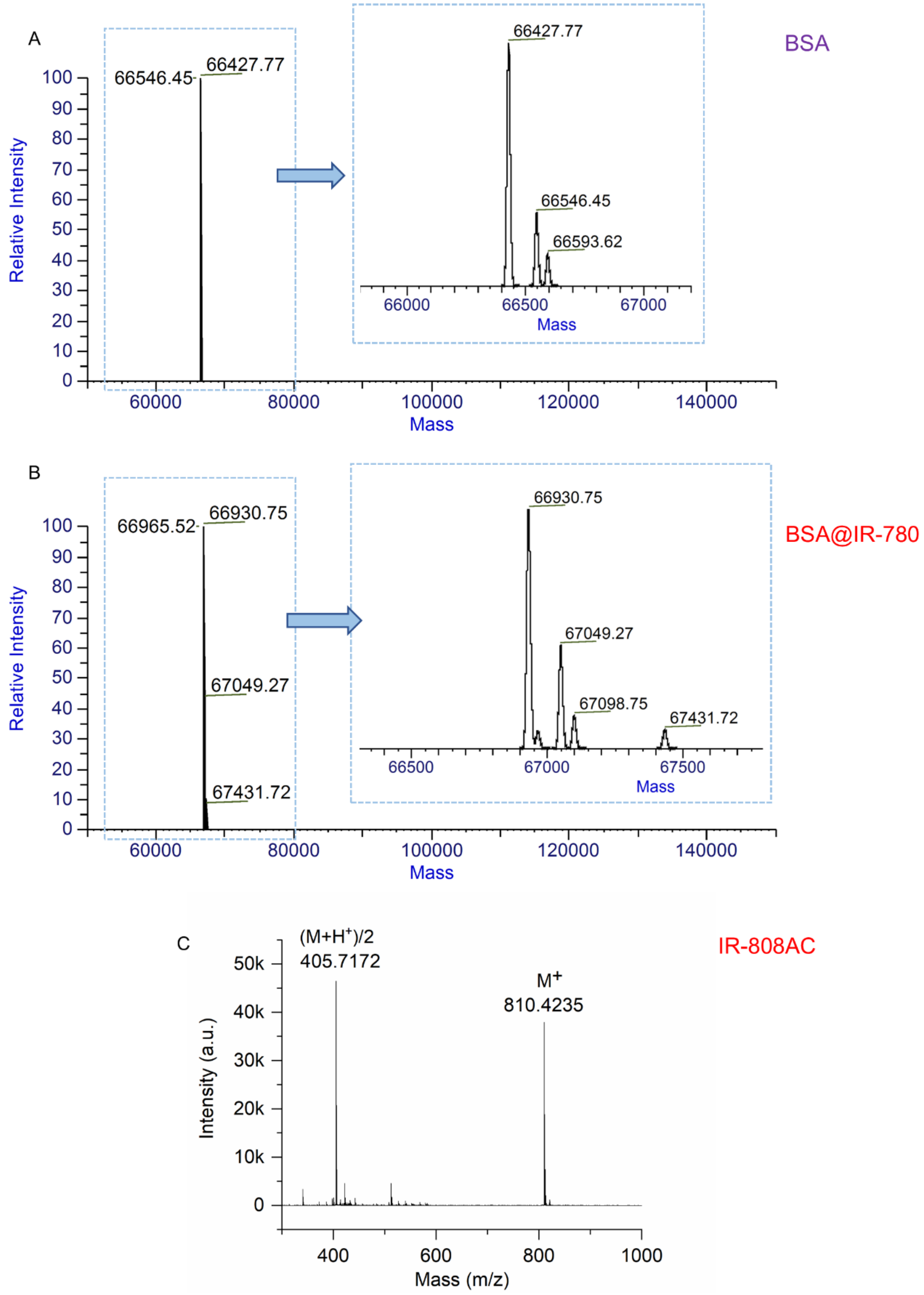

Figure S13. High-resolution mass spectrometry of the free BSA, BSA@IR-780, IR-808AC. (A, B) BSA covalently bound to IR-780. (C) The molecular weight of IR-808AC.

Table S1. Primer sequences for qPCR.

Cytokines name	FORWARD	REVERSE
CCL2	TTAAAAACCTGGATCGGAACCAA	GCATTAGCTTCAGATTTACGGGT
CCL3	TTCTCTGTACCATGACACTCTGC	CGTGGAATCTTCCGGCTGTAG
CCL5	GACACCACTCCCTGCTGCTTTG	CTCTGGGTTGGCACACACTTGG
CXCL1	GGCTGGGATTCACCTCAAGAACATC	TGAGTGTGGCTATGACTTCGGTTTG
CXCL9	TCCTGGAGCAGTGTGGAGTTCG	AATTGGGGCTTGGGGCAAACTG
CXCL10	TGCCTCATCCTGCTGGGTCTG	CATTCTCACTGGCCCGTCATCG
IL-6	CTTCTTGGGACTGATGCTGGTGAC	TCTGTTGGGAGTGGTATCCTCTGTG
β-actin	GAAGTGTGACGTTGACATCCG	GTCAGCAATGCCTGGGTACAT
